# Supplementary material for: Can we do better? A qualitative study in the East of England investigating patient experience and acceptability of using the faecal immunochemical test in primary care
Source: BMJ Open. 2023 Jun 13;13(6):e072359. doi: 10.1136/bmjopen-2023-072359 (PMC10277082; doi:10.1136/bmjopen-2023-072359)
Supplement: Supplementary data [file bmjopen-2023-072359supp001.pdf]

Supplementary File 1. Interview topic schedule

Amended COVID items in italics

Background

- Tell me a bit about you:
  - age
  - education
  - employment history
  - daily activities
  - general health

- *What impact has coronavirus had on your everyday life?*

Background to FIT

- What was your overall experience of the problem, getting tested and receiving your test result?

- Tell me more about the symptoms you experienced
  - Abdominal discomfort
  - Change in bowel habit (general, diarrhoea, constipation)
  - Bleeding
  - Indigestion / heartburn
  - Bloating
  - Fatigue
  - Unexplained weight loss
  - Loss of appetite.

- How long had you experienced symptoms?
  - *Note: Try and get dates to be able to work out how this relates to COVID*

- Did you try any self-management?
  - *If yes, do you think coronavirus affected how you self-managed?*

- When did you first seek help for symptoms?
  - *Do you think coronavirus had any impact on when and where you sought help?*
    - *Friends and family*
    - *Other sources of information (e.g. internet)*
    - *Other medical professional (e.g. pharmacist)*
  - *Do you think coronavirus has changed the way you seek help for medical problems?*
    - *Changing thresholds for going to the doctors*
    - *Ongoing fear of going out*
    - *Change in the way that clinical appointments happen*

- Did you take any prescription medication for symptoms?
  - How long taking
  - Any changes to medication and/or dose
  - How medication has helped
  - Any problems?
  - *Do you think coronavirus led to any problems getting medication?*

Experience of GP consultation where the FIT-poo test was first suggested

- How they were feeling before GP appointment (about symptoms, urgency, what did they expect to happen)?

- *Did coronavirus have any impact on how you interacted with your GP?*
    - *Did you have a face to face appointment?*
    - *Were there any remote part to the consultation?*
    - *Was this what you were expecting?*

- *What was it like to going to the GP practice during coronavirus?*
    - *How did this make you feel?*
    - *How do you feel about attending any future appointments with your GP?*

- How did you feel when the GP suggested having a FIT-poo test?
    - How was the FIT-poo test introduced and explained?.
    - Who explained this (GP, nurse, admin?).
    - What did you think about the information you received (format and content).

- How did you feel following the GP consultation but before doing the test (physically and emotionally)?

- How long did you wait between the GP visit and doing the FIT-poo test at home?
    - What factors influenced this (busy/scared/anxious/collecting additional information?).
    - Did you discuss doing the FIT-poo test with anyone (family member, friend, GP, nurse)
    - What was your experience of discussion?
    - Did you look up or request any further information (including internet).
    - Any concerns about taking the test?

- Do you have any ideas about why people might decide against taking the test (why others might be put off)?

- What did you think the doctor was looking for?
    - Did you or they make any links between colorectal cancer and your these symptoms?

Experience of doing the FIT-poo test

- How did you feel about doing the test?
    - Physically
    - Emotionally
    - Did you manage first time you tried? If not, why not?

- How did you feel following the test?
    - Explore for immediately after test
    - Later that day
    - Following day
    - Over following week.
- *Do you think the coronavirus made you more/less concerned about the test?*

Experience of receiving the result of the FIT-poo test

- What was it like waiting for result?
    - *Do you think coronavirus affected this?*
    - For those who have not yet had a result – why do they think this might be?
    - How did you receive the test result (text message, letter, phone call, another consultation)
    - *Do you think coronavirus has had any impact on the timeliness/delivery of results?*

- What was it like being given a POSITIVE / NEGATIVE / EQIVOCAL result?

- Do you know what will happen next?
    - *Are there any concerns about future care because of coronavirus?*

- Do you think your experience has influenced any of your friends or family members about seeking/receiving care now and in the future?

Improvements to the FIT-poo test experience

- What went well before, during and after doing the FIT-poo test?
  - Any suggestions for changes that would improve FIT-poo test
    - Information provided
    - Procedure
    - Staff interpersonal skills
    - Experience of taking the FIT-poo test.

- *What impact, if any, did coronavirus have on your testing experience?*
  - *Has this changed their view of future care and what this might look like post-covid?*

- Would you take test again?

- Would you recommend test to someone else?
  - How would you explain test to someone else?

Ask whether anything else participant would like to mention / discuss
