# Supplementary material for: Can we do better? A qualitative study in the East of England investigating patient experience and acceptability of using the faecal immunochemical test in primary care
Source: BMJ Open. 2023 Jun 13;13(6):e072359. doi: 10.1136/bmjopen-2023-072359 (PMC10277082; doi:10.1136/bmjopen-2023-072359)
Supplement: Supplementary data [file bmjopen-2023-072359supp002.pdf]

# Supplementary File 2. Interviews in context: patients' experiences during the COVID-19 pandemic

Participants described feeling isolated from their support networks due to the limitations on social interactions during the pandemic. This resulted in some feeling unable to have conversations with family and friends regarding their symptoms during the appraisal period.

Many delayed seeking help as a result of what they perceived was happening within General Practice. A range of attitudes were displayed, from believing practices were overrun, to feeling GPs were 'shutdown' or only open for emergencies. For some, this resulted in the decision to seek help from an alternative provider such as 111 or walk-in services. In contrast, for a minority of participants positive media campaigns advising the public that the NHS remained open influenced them to contact their GP.

Changes to remote consulting (primarily in the form of telephone consultations) had both positive and negative impacts. Though many highlighted the convenience of telephone appointments and the speed of talking to their GP, others described the challenges of navigating complicated booking systems, and describing problems over the phone.

For those that visited their GP surgery during the pandemic, personal protective equipment and social distancing measures were generally expected and accepted, despite often feeling time-consuming or frustrating. Many perceived GP practices to be safer and cleaner than other public spaces.
