# Supplementary material for: Can we do better? A qualitative study in the East of England investigating patient experience and acceptability of using the faecal immunochemical test in primary care
Source: BMJ Open. 2023 Jun 13;13(6):e072359. doi: 10.1136/bmjopen-2023-072359 (PMC10277082; doi:10.1136/bmjopen-2023-072359)
Supplement: Author's manuscript [file bmjopen-2023-072359.draft_revisions.pdf]

BMJ Open

BMJ Open is committed to open peer review. As part of this commitment we make the peer review history of every article we publish publicly available.

When an article is published we post the peer reviewers’ comments and the authors’ responses online. We also post the versions of the paper that were used during peer review. These are the versions that the peer review comments apply to.

The versions of the paper that follow are the versions that were submitted during the peer review process. They are not the versions of record or the final published versions. They should not be cited or distributed as the published version of this manuscript.

BMJ Open is an open access journal and the full, final, typeset and author-corrected version of record of the manuscript is available on our site with no access controls, subscription charges or pay-per-view fees (http://bmjopen.bmj.com).

If you have any questions on BMJ Open’s open peer review process please email info.bmjopen@bmj.com

BMJ Open

Can we do better? A qualitative study investigating patient experience and acceptability of using the faecal immunochemical test in primary care

Journal:	*BMJ Open*
Manuscript ID	bmjopen-2023-072359
Article Type:	Original research
Date Submitted by the Author:	01-Feb-2023
Complete List of Authors:	Snudden, Claudia; University of Cambridge Department of Public Health and Primary Care,<br>Calanzani, Natalia; University of Aberdeen Institute of Applied Health Sciences, Academic Primary Care; University of Cambridge Department of Public Health and Primary Care<br>Archer, Stephanie; University of Cambridge Department of Public Health and Primary Care<br>Honey, Stephanie; University of Leeds Leeds Institute of Health Sciences<br>Pannebakker, Merel; University of Cambridge Department of Public Health and Primary Care<br>Faher, Anissa; University of Cambridge School of Clinical Medicine<br>Chang, Aina; University of Cambridge School of Clinical Medicine<br>Hamilton, Willie; University of Exeter<br>Walter, Fiona M; Queen Mary University of London, Wolfson Institute of Population Health; University of Cambridge, Department of Public Health and Primary Care
Keywords:	PRIMARY CARE, QUALITATIVE RESEARCH, GASTROENTEROLOGY

SCHOLARONE™
Manuscripts

I, the Submitting Author has the right to grant and does grant on behalf of all authors of the Work (as defined in the below author licence), an exclusive licence and/or a non-exclusive licence for contributions from authors who are: i) UK Crown employees; ii) where BMJ has agreed a CC-BY licence shall apply, and/or iii) in accordance with the terms applicable for US Federal Government officers or employees acting as part of their official duties; on a worldwide, perpetual, irrevocable, royalty-free basis to BMJ Publishing Group Ltd ("BMJ") its licensees and where the relevant Journal is co-owned by BMJ to the co-owners of the Journal, to publish the Work in this journal and any other BMJ products and to exploit all rights, as set out in our licence.

The Submitting Author accepts and understands that any supply made under these terms is made by BMJ to the Submitting Author unless you are acting as an employee on behalf of your employer or a postgraduate student of an affiliated institution which is paying any applicable article publishing charge ("APC") for Open Access articles. Where the Submitting Author wishes to make the Work available on an Open Access basis (and intends to pay the relevant APC), the terms of reuse of such Open Access shall be governed by a Creative Commons licence – details of these licences and which Creative Commons licence will apply to this Work are set out in our licence referred to above.

Other than as permitted in any relevant BMJ Author's Self Archiving Policies, I confirm this Work has not been accepted for publication elsewhere, is not being considered for publication elsewhere and does not duplicate material already published. I confirm all authors consent to publication of this Work and authorise the granting of this licence.

For peer review only

Can we do better? A qualitative study investigating patient experience and acceptability of using the faecal immunochemical test in primary care

Claudia M Snudden[1] BMBCh (ORCID ID: 0000-0002-7355-4317)
Natalia Calanzani[1,2] PhD (ORCID ID: 0000-0002-5068-2543)
Stephanie Archer[1] PhD (ORCID ID: 0000-0003-1349-7178)
Stephanie Honey[3] PhD (ORCID ID: 0000-0002-2675-7824)
Merel M Pannebakker[1] PhD (ORCID ID: 0000-0003-2918-0570)
Anissa Faher[4] BA Hons (Cantab)
Aina Chang[4] BA Hons (Cantab)
Willie Hamilton[5] FRCGP (ORCID ID: 0000-0003-1611-1373)
Fiona M Walter[6,1] FRCGP (ORCID ID: 0000-0002-7191-6476)

[1] The Primary Care Unit, Department of Public Health and Primary Care, University of Cambridge, Cambridge, UK
[2] Academic Primary Care, Institute of Applied Health Sciences, University of Aberdeen, Aberdeen, UK
[3] Leeds Institute of Health Sciences, University of Leeds, Leeds, UK
[4] School of Clinical Medicine, Addenbrooke's Hospital, University of Cambridge, UK
[5] University of Exeter Medical School, Exeter, UK
[6] Wolfson Institute of Population Health, Queen Mary University of London, UK

Corresponding author: Dr Claudia Snudden
Email: cs2048@medschl.cam.ac.uk

Address: The Primary Care Unit, Department of Public Health and Primary Care, University of Cambridge, Cambridge, UK

Word count: 4388

For peer review only

ABSTRACT

Objectives

The faecal immunochemical test (FIT) is increasingly used in UK primary care to triage patients presenting with symptoms and at different levels of colorectal cancer risk. Evidence is scarce on patients' views of using FIT in this context. We aimed to explore patients' care experience and acceptability of using FIT in primary care.

Design

A qualitative semi-structured interview study. Interviews were conducted via Zoom between April and October 2020. Transcribed recordings were analysed using framework analysis.

Setting

East of England General Practices.

Participants

Consenting patients (aged ≥40 years) who presented in primary care with possible symptoms of colorectal cancer, and for whom a FIT was requested, were recruited to the FIT-East study. Participants were purposively sampled for this qualitative sub-study based on age, gender, and FIT result.

Results

44 participants were interviewed with a mean age 61 years, and 25 (57%) being male: 8 (18%) received a positive FIT result. Three themes and seven subthemes were identified. Participants' familiarity with similar tests and perceived risk of cancer influenced test experience and acceptability. All participants were happy to do the FIT themselves and to recommend it to others. Most participants reported that the test was straightforward, although some considered it may be a challenge to others. However, test explanation by healthcare professionals was often limited. Furthermore, while some participants received their results quickly, many did not receive them at all with the common assumption that "no news is good news". For those with a negative result and persisting symptoms, there was uncertainty about any next steps.

For peer review only

Conclusions

While FIT is acceptable to patients, elements of communication with patients by the healthcare system show potential for improvement. We suggest possible ways to improve the FIT experience, particularly regarding communication about the test and its results.

For peer review only

STRENGTHS AND LIMITATIONS OF THIS STUDY

- Study describes rich data on patients' care experiences and acceptability when FIT is used primary care – generating essential evidence as FIT use has intensified in this setting
- Interested participants were contacted within four weeks of performing FIT, helping to reduce recall bias
- All participants were White (British or other) and English speakers – views may differ for those identifying with other ethnicities or who do not understand English well
- The use of Zoom for participant interviews may have hindered those unable to navigate the required technology from taking part

For peer review only

INTRODUCTION

Most patients diagnosed with colorectal cancer (CRC) in the UK will first present to their General Practitioner (GP) with abdominal symptoms[1,2]. However, diagnosing CRC, particularly in its early stages, can be difficult as symptoms of CRC are broad and non-specific[3]. GPs in England currently follow NICE 2015 guidelines for patients with suspected cancer to identify higher risk patients who should be urgently referred for further investigation[4]. In 2017, NICE recommended the use of the Faecal Immunochemical Test (FIT) within primary care to help triage low risk patients with possible CRC symptoms that do not meet urgent referral criteria[5,6]. FIT can be used for both ruling in and ruling out CRC[7,8]. Alongside its use in symptomatic patients, FIT is also used in the UK's bowel cancer screening programme, having replaced the previously used guaiac faecal occult blood test (gFOBT)[9].

The threshold set to define a positive FIT result (i.e., the haemoglobin cut-off concentration) differs between populations. For symptomatic patients, NICE advises that those with a FIT result ≥ 10µg Hb/g faeces are referred on for further diagnostic testing; of these about 7% will be diagnosed with CRC[5,7]. In contrast, England uses a cut-off of 120µg Hb/g in asymptomatic screening to select who requires further investigation[9]. Variations in thresholds are due to the differences in risk between asymptomatic and symptomatic populations; the risk of cancer is higher for those with symptoms therefore the threshold for further investigations is lower.

As a result of the COVID-19 pandemic, FIT use in primary care was intensified to help prioritise urgent referrals[10–13]. In June 2022, The British Society of Gastroenterology released additional guidelines recommending the continued use of FIT as a diagnostic triage tool for all urgent referrals[14,15]. NICE also plans to publish additional guidance on FIT by November 2023[16]. Given the increasing reliance of FIT use in primary care, it is critical to understand what patients think of the test and whether they find it acceptable.

Studies conducted in screening contexts have shown that disgust associated with performing stool tests and certain procedural aspects of FIT can present barriers to uptake, however, the test is often found acceptable[17–22]. Nonetheless, there is still limited research into FIT experience and acceptability, particularly within the context of symptomatic patients[23]. Given this group of individuals have varied (and higher) levels of risk compared to an asymptomatic screening population, evidence on the patients' experience of care when and after doing FIT is crucial. Increased use of FIT is likely to result in the need for more safety-netting so that

For peer review only - http://bmjopen.bmj.com/site/about/guidelines.xhtml

opportunities to detect cancer are not missed in those with negative results and persisting symptoms.

This study aims to bridge these gaps, exploring FIT experience and acceptability for patients presenting to primary care with possible symptoms of CRC.

For peer review only

METHODS

Design, setting and population

This qualitative sub-study was undertaken as part of the FIT-East study, which was set across General Practices in the East of England Cancer Alliance (Suffolk & North East Essex and Norfolk & Waveney)[24]. FIT-East included 507 patients aged 40 years and older with possible symptoms of CRC, but who did not immediately meet urgent referral criteria, for whom a FIT was requested by the GP[24]. FIT kits contained a study recruitment letter and consent form; participants could tick a box if interested in taking part in interviews.

We undertook semi-structured interviews with patients to investigate experience and acceptability of using FIT in primary care. Interviews were also carried out with General Practitioners (reported elsewhere[25]). Ethical approval was granted by the East of England Cambridge Central Research Ethics Committee – reference 19/EE/0036.

Sampling

We undertook purposive sampling based on age, gender, and FIT result to obtain a range of experiences among patients who consented to take part in the FIT-East study. Potential participants were contacted by email (provided by patients in the consent forms). To reduce recall bias, we contacted patients within four weeks of FIT being sent to the laboratory.

Ninety-nine participants responded positively to the initial email and were invited for interview. Additional informed consent was required and obtained for all patients. Interviews were carried out until no new topics were discussed: 45 participants were interviewed, mean duration 39.5 minutes. Due to the COVID-19 pandemic, interviews were carried out remotely using the Zoom platform, and audio-recorded. One interview could not be analysed due to a technical fault during recording; therefore 44 participants were included. Each participant was individually interviewed once by one or two experienced female health services researchers with a background in Psychology (SA and MMP) or Nursing (SH).

Interview topic guide

The guide (Supplementary File 1) explored patient experiences of being asked to undertake a FIT in primary care, covering areas including: obtaining the test, using it, returning it, and receiving the results. The topic guide was piloted by the research team before use. In order to

For peer review only - http://bmjopen.bmj.com/site/about/guidelines.xhtml

better understand patients' experiences during the COVID-19 pandemic, additional questions were added in June 2020. Interviews were carried out between April and October 2020.

Data analysis

Verbatim transcripts were checked, anonymised and analysed using framework analysis[30]. Four researchers (one academic GP [CS], one health services researcher [NC], and two medical students [AF and AC]) repeatedly read the transcripts and coded the interviews. Coding was informed by the Model of Pathways to Treatment[26,27], the Theoretical Framework of Acceptability (TFA)[28] and Forster's *et al* considerations when assessing acceptability of diagnostic tests[29], in order to place findings within the context of the cancer diagnostic pathway and patient's experiences of care. Eight interviews were initially coded inductively by CS and an analytical framework developed. The framework was then used to systematically index the remaining transcripts, including double coding of a quarter of the data set. Codes and categories were constantly refined during analysis. Data were charted into a series of matrices from which themes were developed. Consistency of coding was discussed regularly during team meetings; themes were also amended with guidance from senior team members (SA and FMW). NVivo 12 was used to facilitate analysis[31].

Participant age (range), gender (M for male and F for female) and FIT results (positive, negative or unclear) are reported alongside quotes to aid interpretation of findings. Quotes were chosen to illustrate findings while also ensuring a good spread of participants and their characteristics.

Patient and public involvement

Patient-public partners from the CanTest Collaborative[32] (responsible for FIT-East) provided feedback on the original and amended interview topic guides. Representatives also read and commented on interview transcripts; these comments were considered when iteratively revising our analysis.

For peer review only - http://bmjopen.bmj.com/site/about/guidelines.xhtml

RESULTS

Table 1 describes characteristics of the included participants and describes how FIT results were received. About a fifth (18%) had a positive FIT result. Outcome data for whether participants received a cancer diagnosis was not available.

Table 1. Demographic characteristics and FIT results (n=44)

Characteristic	Variable	N	%
Sex	Female	19	43
	Male	25	57
Age (years)	40-49	3	7
	50-59	17	39
	60-69	10	23
	70-79	11	25
	80-89	3	7
FIT result	Negative	35	80
	Positive	8	18
	Unclear*	1	2
How FIT result was received	Patient called practice†	12	27
	Patient did not receive results/is unsure‡	11	25
	Practice called patient§	9	20
	Patient told face-to-face‖	4	9
	Patient received letter¶	3	7
	Patient checked results online¶	2	5
	Other**	3	7
Education level	None	3	7
	GCSE (or equivalent)	4	9
	A level (or equivalent)	5	11
	Degree (or equivalent)	18	41
	Other higher education	10	23
	Missing	4	9
Ethnicity	White British	35	80
	White Irish	1	2
	Any other White background	2	4
	Missing	6	14
IMD quintiles	1 (most deprived)	0	0
	2	4	9
	3	7	16
	4	4	9
	5 (least deprived)	6	14
	Missing	23	52

	* One patient described doing the FIT in detail, but on the patient database FIT was recorded as "not done". As the patient clearly had done the test, this was recorded as an unclear result.<br>†One positive result (patient reported this as inconclusive during the interview).<br>‡Did not receive (n=7, all negative), unsure about it (n=4, 2 negative, one positive and one missing)<br>§GP (n=6, 2 positive results), nurse practitioner (n=1, negative – nurse called about something else and the patient asked for results); receptionist (n=2, 1 positive result).<br>‖GP (n=2, all negative), nurse (n=1, positive), receptionist (n=1, negative)<br>¶ All negative results<br>**Cannot remember how results were received (n=1, positive), GP contacted but unclear if by phone or face-to-face (n=1, negative), over the phone but unclear who called whom (n=1, positive).

Overview and Context

We identified three themes and seven subthemes describing patient experience and acceptability of using FIT after presenting in primary care with possible symptoms of CRC (but not meeting immediately criteria for urgent referral) (Figure 1).

As the study was performed during the COVID-19 pandemic a brief description of COVID-19 related themes is available for context (Supplementary file 2). The pandemic did not appear to influence participants' experiences of using FIT, but it did affect how participants accessed their GP prior to receiving the test.

The role of personal factors on participant's views

Perceived risk of cancer due to personal/family history or experience

A personal history of having had cancer in the past encouraged participants to seek help quickly for their symptoms. Similarly, cancer experiences from family and friends influenced their decision to seek help after noticing body changes, and to do the test when it was offered:

> *"My father has had bowel cancer. He basically had it once and they took a section of the colon out. After a few years it returned so they removed the whole of his colon and he's now been…you know, God bless he's still alive and it's been sort of over 15 years he's been clear but there is that sort of history that I was aware of. So I thought, alright, better…you know, because it had changed slightly, yes, and it was bleeding more often, I thought I'd better go and get it looked at."* (PT18, M, 50-59y, negative)

Some participants became concerned that their symptoms could be a sign of bowel cancer - either from their own knowledge of 'red flag' symptoms or from reading about symptoms online, leading them to actively seek advice. Others became increasingly anxious or concerned about

For peer review only - http://bmjopen.bmj.com/site/about/guidelines.xhtml

the severity of their symptoms, particularly if things had worsened over time or were causing significant discomfort.

On the other hand, having had a relatively recent examination or negative investigation often reassured them there was nothing serious and being offered a FIT was not considered to be worrying or concerning:

> *"No, because I'd had that colonoscopy, and that they'd told me I'd got the diverticular disease; so I thought I was, you know, and there was no sign for cancers then so I thought, 'well, I'm okay'"* (PT4, F, 70-79y, unclear result)

Familiarity from screening and previous tests

Many participants were familiar with doing stool tests, usually as part of bowel screening or previous diagnostic processes. As a result, many displayed no concerns with the idea of doing FIT:

> *"Well, I've been doing them for years. It didn't upset me, if that's what you mean. It's just one more thing, going to the bog isn't it? Going to the loo? Except this time you put it on a piece of […] paper, and get a smear and stick it in the packet, and then seal it up."* (PT1, M, 70-79y, negative)

A few who had had previous experiences within secondary care (such as colonoscopies due to diverticular disease) described the FIT experience as less daunting:

> *"I was quite relaxed about* [being asked to do FIT] *because I'd had a colonoscopy before, that was five years ago, so that was more nerve-racking."* (PT25, F, 60-69y, negative)

Overall acceptability of FIT

Happy to be offered FIT, do it and recommend it to others

For peer review only - http://bmjopen.bmj.com/site/about/guidelines.xhtml

Most participants reported they were glad or relieved to be offered FIT and accepted the need to do it. This was true even when the potential unpleasantness or difficulties of doing a FIT were recognised. For some, acceptance was associated with wanting to find an answer to their symptoms, especially if these had been particularly distressing:

> *"I guess I felt a little bit strange, kind of, like here I am, you know, lining the toilet to catch my stools. But when you know that it's something that you want to get done and want to get answers for you just get on and do it really."* (PT12, F, 40-49y, negative)

For others, doing a test could lead to an 'earlier diagnosis' and the potential to start treatment sooner if there was something serious going on:

> *"So therefore if you have any problems, it's always best to get it checked out, so yeah, that's what I was thinking, if there was anything wrong, I would rather know now than leave it and it spreads."* (PT9, F, 50-59y, negative)

When reflecting on why *others* might not want to do FIT, common reasons included the fear of a potential cancer diagnosis and finding the process disgusting or embarrassing:

> *"Probably because it's not a very nice thing to have to do, you know. Playing around with poo is not something that everybody thinks, 'oh that sounds like fun'."* (PT3, F, 60-69y, negative)

Every participant reported being happy to repeat FIT if necessary, and all would willingly recommend FIT to others if they needed it.

FIT was straightforward despite challenges – others may struggle

At least half the participants commented on challenges they faced when physically collecting the FIT sample: often these were difficulties writing the label, 'catching' the stool sample without it falling into the toilet water or when suffering with diarrhoea, and finding the FIT stick small or *"very fiddly"* to handle.

Several participants expressed anxiety about taking the FIT sample and 'wanting to do it right':

For peer review only

For peer review only - http://bmjopen.bmj.com/site/about/guidelines.xhtml

> *"Only that I didn't overload it because I think it's…, you know, kind of like it shows you the picture and it's just, don't do this, and do this, and you're like, 'oh my God but how much is too much, and how much is too little'? […] So I think, that was my only thing was that I wanted to do it right and I didn't want to have to do it again.*" (PT5, F, 50-59y, positive)

Many reported wanting to get the sample done and feeling relieved after completing the test:

> *"I wanted to get it out of the way, it's not exactly difficult, but it was just the having to, you know, deal with it. So I woke up thinking, 'oh, here we go, you know, I need to do this' and when I'd got things in the specimen jars and I sorted myself out, I was glad it was over."* (PT23, F, 50-59y, negative)

Several participants described methods to overcome difficulties collecting the sample, largely involving finding a suitable collecting container. For many, this drew on prior experiences when completing bowel cancer screening tests. Nonetheless, most participants (even some reporting these challenges) were confident about their ability to do the test, often remarking how 'straightforward' FIT was:

> *"In terms of the actual doing the test, I don't see that you could make it any simpler or easier really, to be honest."* (PT36, F, 50-59y, negative)

In particular, participants found the instructions generally easy to follow. Nonetheless, they discussed why *others* might struggle with FIT – from potential difficulties faced by having arthritic or shaky hands, poor eyesight, or an upset stomach, to issues following instructions:

> *"I would worry about the size of the wand thing for people whose hands are a bit more crippled or who can't hold things so well."* (PT28, F, 70-79y, positive)

It was not explained well, but…

Most participants agreed that the explanations given by their healthcare professional (usually a GP or practice nurse) regarding what FIT was and why it was indicated, tended to be poor:

For peer review only - http://bmjopen.bmj.com/site/about/guidelines.xhtml

"My GP didn't, actually, tell me what it was. I had to Google it to even find out what it was. He said 'we'll leave you out a FIT test', and I had no idea what it was." (PT14, M, 60-69y, positive)

A few participants defended the professionals in such situations, attributing the limited information to common constraints in a consultation:

"It wasn't her [GP] fault, 'cause I'd actually asked her to do two medical problems on the same appointment, so she had to do it a bit rushed." (PT16, M, 50-59y, negative)

Often participants were asked to do multiple stool tests as part of their symptom work-up, leading to confusion regarding the differences between tests. Frequent references were made to being handed a bag containing a FIT and being told to just follow the instructions. For two participants, the poor communication even resulted in confusion of the test with the similarly named Fitbit watch:

"[I]t wasn't really explained to me, she just gave me this bag and said, 'right, well we'll do two blood tests, we'll do a bowel test and then we've got this other one called FIT Bit' and she just gave me a bag and she said 'it's all in there'." (PT38, F, 60-69y, negative)

Whilst the text explanation was often inadequate, some participants had very good intuition about linking FIT to CRC. Some reported that their GP had alluded to cancer but did not use the word 'cancer' specifically, choosing instead to describe it as "*something sinister*" or "*something nasty lurking*". Despite this, many appeared to interpret the GP's language appropriately:

"When he suggested doing a FIT test, which he explained to me what it was, I thought 'yeah, it makes sense really because I know what you're after'. He didn't tell me what he was after, but I thought 'I know what you're looking for'." (PT24, M, 50-59y, negative)

Participants' understanding of how FIT worked varied considerably. The most common explanations focused around looking for traces of (often non-visible) blood in the stool, but others described FIT as "*a bit of an MOT check*", being used to "*determine the health of the gut*", "*check the digestive system is working properly*" or look for "*abnormalities within the poo*". A few participants used the internet to further research FIT and causes of blood in the stool.

Whilst most participants demonstrated good intuition or a basic understanding of FIT, some had no awareness as to why they were asked to do FIT, yet did it anyway:

For peer review only - http://bmjopen.bmj.com/site/about/guidelines.xhtml

"I just got given the test to do and, I suppose, in a roundabout, sort of, way, she was saying, you know, 'you need to have the test done', but I didn't understand it and I was puzzled about it [...] I think that's probably why I kept reading the notes about it, because I kept thinking, 'well, I'm not sure why I'm doing this'." (PT33, F, 50-59y, negative)

Several participants talked about trusting the GP and their advice, and how this influenced their decision to do the FIT, how it would affect their decision to do it again or recommend it to others:

"I like her, I think she's an excellent GP. If she asks me to do something, to participate or provide a test for example, I trust her to be asking that for a good reason and I will instinctively comply with any requests for tests or anything else as I'm assuming that it's going to provide information, test results or whatever which will be for my direct benefit." (PT19, M, 60-69y, negative)

Communicating FIT results and follow-up

Uncertainty regarding FIT results

Participants reported contrasting attitudes while waiting for test results. Several discussed the anxiety surrounding the waiting period, at times hypothesising what would happen next in the worst-case scenario:

"Oh, I think just a bit anxious really. As though…you're bound to be, and yes, just a bit anxious and just waiting for the results and then oh, what's going to happen next?" (PT28, F, 70-79y, positive)

In contrast, many were unaffected by waiting, seemingly managing to *"forget about it"* and carry on with life as usual. This was often linked to either normalising FIT to a more 'standard' test, or expecting to receive a negative result and therefore not seeing reason to worry:

"Well, I wasn't anxious about it. To me, it's just like having a blood test. You know, I wasn't sitting around wondering, 'well when will I get the result and what will it be'; it was just another test." (PT39, M, 80-89y, negative)

For peer review only - http://bmjopen.bmj.com/site/about/guidelines.xhtml

Many participants felt they weren't given clear information by the GP on when and how to access their results. Some did not receive their results at all, others contacted the practice for these. Many assumed that the practice would only contact them if there was a positive result and reported not being worried as *"no news is good news"*:

> *"I haven't actually been told anything so I presume they were negative or there were no concerns because no GP has contacted me."* (PT20, F, 50-59y, negative)

Some participants felt that chasing results should be practice-led rather than patient-led, regardless of the result:

> *"Only that it would be good if the doctors surgery rang up to say it was normal, but I suppose they have so many phone calls and things to make that they just don't ring you up if it's normal."* (PT30, F, 50-59y, negative)

Receiving FIT results and next steps

For those who received their test results, there were often comments on receiving these quickly. Nonetheless, the way results were communicated was not always well received, both for positive and negative results:

> *"The receptionist at the GP surgery telephoned me and said the doctor had referred me[...] and I said, 'oh, why is that then', and she...she didn't seem to know and I said 'I just had a FIT test, what was the result of that'? Pause, and she said, 'oh, yes, it was positive. I expect that's why it is'. And I'm not joking, that's exactly how the conversation went.*" (PT14, M, 60-69y, positive)

> *"I think one slightly disappointing aspect I suppose is that when I phoned for results, I only spoke to the receptionist, and all they were able to tell me was no further action necessary. And I kind of thought, 'oh well, it's great in one way and I was pleased with that, but it kind of didn't explain why I'd had problems earlier' and I thought, 'oh, do I just wait to see if I ever have this problem again'."* (PT41, M, 50-59y, negative)

For participants with a positive result, there were reported delays in having a colonoscopy, particularly during the COVID-19 pandemic. Participants were often stoical about this situation.

For peer review only - http://bmjopen.bmj.com/site/about/guidelines.xhtml

For peer review only

For those with a negative result, a sense of relief and reassurance was frequently reported. However, when symptoms persisted, there was uncertainty regarding what to do next. Some said they would have valued a follow up consultation with their GP, but in practice this rarely happened, leaving participants feeling their problems had not been fully resolved. Others believed that the GP would not be able to offer them anything further even if they did arrange a further appointment:

> *"No, I don't know if there's a follow-up or anything, if you detect nothing does it just get swept under the carpet or do I stay on your database and do you check up again or…I don't know?"* (PT6, M, 60-69y, negative)

For peer review only

DISCUSSION

Summary

This study of patients presenting in primary care with symptoms of possible CRC but not immediately meeting criteria for urgent referral found FIT an acceptable and straightforward test, despite some challenges manipulating the test kit. Participant characteristics such as past test experiences and perceived personal risk of cancer influenced attitudes towards FIT. Participants were also happy to do FIT again and recommend it to others, and trusted that their GP knew what was best for them. On the other hand, test explanations from health professionals were generally poor. This could be due, in part, to the lack of clarity surrounding FIT implementation and diagnostic pathways within primary care during the COVID-19 pandemic. There was also reported dissatisfaction with the way results were received, for both positive and negative results. Many participants did not receive results at all, and several assumed that "*no news is good news*". For some, there was no follow-up after a negative result, even when symptoms persisted.

Strengths and limitations

To our knowledge, this is the first qualitative study to explore experience and acceptability of FIT for symptomatic patients. Three well-established theoretical models were used to guide the analysis[26–29]. We found that the models complemented each other, with overlap between different elements.

Interested participants were contacted within four weeks of performing FIT, helping to reduce recall bias. There was good variation amongst participants in terms of age, gender and FIT result. However, only a sample of patients who took part in the FIT-East study were interviewed; views of those who did not participate in the interviews may differ. The sample was also limited to the East of England. All participants identified themselves as White, slightly higher than the proportion reported by the UK Census for this region[33], and understood English well. Experiences of patients identifying with other ethnicities, and of those who do not speak English well may differ and need to be further investigated.

Due to resulting restrictions from the pandemic, interviews were conducted over Zoom, hindering those unable to navigate the required technology from taking part. Finally, no patients aged under 40 were recruited (as defined by our inclusion criteria). As FIT becomes more

For peer review only

widely used in primary care, it will be key to explore the attitudes of this group, less familiar with stool testing and without involvement in prior screening programmes – particularly as the incidence of CRC is increasing in the under 50s[34–36].

Comparison with existing literature

Consistent with existing literature (although mostly investigating asymptomatic populations), FIT is seen as relatively straightforward with clear instructions[19–21,23]. Nonetheless, similar challenges to collecting the sample were reported including difficulties writing the label and handling the FIT stick[20,22]. Familiarity with stool tests is associated with higher intentions to use related tests in future, aligning with the confidence displayed by participants with experience of screening[23]. Social influences have also been associated with encouragement of FIT uptake, impacting an individual's perceived risk of, or susceptibility to, cancer[19,37].

Participants displayed different levels of proactivity in seeking FIT results, reflective of behaviours seen in the literature[38]. Patients reported wanting further clarification on how results are communicated; existing uncertainties were anxiety-inducing at times[20,38,39]. To our knowledge, there is no other available evidence on follow-up (or lack thereof) of patients with persisting symptoms and a negative FIT result.

Implications for research, practice and policy

Building on our findings and published literature, we have developed recommendations for research, practice and policy to improve both the FIT experience and patient safety (Box 1). Rationale for these recommendations is described below.

While FIT acceptance was high, there are still opportunities for improvement, particularly regarding processes to collect the stool sample, and better understanding FIT acceptability across different population groups.

Reports of questionable care about explaining the test, providing results and following up are also concerning[40]. While participants still found FIT acceptable and trusted that the GP knew what was good for them, care experiences caused anxiety to patients and indicate risks regarding continuity of care. Time waiting for results did not seem to be a problem for those who got them; but many reported that they never received the results. While no news was indeed often good news, some patients who chased their results found out they were positive. It is vital

For peer review only - http://bmjopen.bmj.com/site/about/guidelines.xhtml

that GPs are clearer about when results are expected and how to access them, and safety-netting measures are in place to avoid missing cancers and provide better support to patients.

Finally, our results can support policy makers regarding FIT use and implementation within primary care. While flexibility and adaptability are required in the provision of services so different needs can be met[41], it is vital that national policies define standards on acceptable levels of service, particularly regarding how and when information on test results are provided.

Box 1. Recommendations for practice, research and policy

Research

- Investigate 1) challenges in collecting the stool sample, particularly for older people and those with mobility issues; and 2) improvements to sample collection (such as providing a collection container) and instructions (such as clarification on what to do in the case of diarrhoea).
- Investigate whether patients' presenting symptoms (high vs low risk) influence their perception of FIT, as we found that perceived risk and susceptibility influenced acceptability
- Seek to understand the views of patients who choose not to complete FIT after being offered it, to determine 1) why FIT may not be acceptable to some and 2) whether reasons align with issues identified in this study (e.g., test burden and limited information).

Practice

- Work to improve GP communication with patients regarding why FIT is being done, what it looks for, and what a positive result means. Clear communication can also concurrently reduce patient confusion when multiple tests are requested simultaneously.
- Consider implementing a specific time slot for calling for results and promote these changes to patients through information posters around the surgery and batch SMS message updates.
- Specify requirements for improved training of reception staff in handling results. This would include guidance on communicating potentially sensitive information.
- Capitalise on the growth in use of patient messaging platforms such as Accurx[42], resulting from the COVID-19 pandemic, to develop formal pathways within General Practices for pro-actively communicating negative results and safety-netting advice:
  - Encourage direct SMS messaging of negative results to patients using pre-built templates which also contain information advising patients to contact their GP again if symptoms persist.
  - For those without a recorded mobile phone number, adopt a receptionist-led system of contacting the patient's landline, sending a letter informing them of their negative result if unable to get through.

Policy

- Instigate policy nationally to support standardisation of practices, such as those suggested above, within primary care.
- Ensure patients are included within the design and evaluation of future services and care models.

For peer review only

For peer review only - http://bmjopen.bmj.com/site/about/guidelines.xhtml

Figure legend

Figure 1. Themes and subthemes: patient experience and acceptability of using FIT

Acknowledgements

We thank all the patients who agreed to be interviewed. We thank the North East Essex and Suffolk Pathology Services at the East Suffolk and North Essex NHS Foundation Trust, and the Clinical Biochemistry section of Laboratory Medicine at Norfolk and Norwich University Hospitals NHS Foundation Trust. They have added a study recruitment letter, consent form and prepaid envelope to each FIT kit, distributed these to GP practices, and provided data on FIT results. We thank Prof Niek de Wit and Dr Peter Holloway for their support in the earlier stages of the study. We are grateful to Dr Marije van Melle for her work in the early stages of study design. We thank James Brimicombe for data management advice, support and expertise, and Andy Cowan for his help collecting and managing FIT-East study data.

Supplemental material

Supplementary File 1. Interview topic schedule
Supplementary File 2. Interviews in context
COREQ checklist

Patient consent for publication

Not required.

Funding statement

This work was supported by the CanTest Collaborative, which is funded by Cancer Research UK grant number C8640/A23385, of which FMW and WH are co-Directors, and NC, MMP and SH are postdoctoral researchers.

This work was co-funded by the National Institute for Health Research (NIHR) Policy Research Programme, conducted through the Policy Research Unit in Cancer Awareness, Screening and Early Diagnosis, PR-PRU-1217-21601. The views expressed in this publication are those of the authors and not necessarily those of the National Health Service, the NIHR or the Department of Health. The funding sources had no role in the study design, data collection, data analysis, data interpretation, writing of the report or in the decision to submit for publication.

Competing interests

None declared.

For peer review only - http://bmjopen.bmj.com/site/about/guidelines.xhtml

Contributors

MM and FMW planned the original study; WH and FMW secured funding. SH, SA and MMP carried out the interviews. Analysis was planned and carried out by CS, NC, SH, SA, AF, AC and FMW. CS and NC drafted the manuscript with senior input from SA and FMW and revised it after critical reviews from all authors. All authors read and approved the final manuscript.

Data sharing statement

Anonymised data are available upon reasonable request.

Ethics Approval Statement

Ethical approval was granted by the East of England Cambridge Central Research Ethics Committee – reference 19/EE/0036.

For peer review only

REFERENCES

1. Koo MM, von Wagner C, Abel GA, McPhail S, Hamilton W, Rubin GP, et al. The nature and frequency of abdominal symptoms in cancer patients and their associations with time to help-seeking: evidence from a national audit of cancer diagnosis. J Public Health Oxf Engl. 2018 Sep;40(3):e388–95.

2. Moreno CC, Mittal PK, Sullivan PS, Rutherford R, Staley CA, Cardona K, et al. Colorectal Cancer Initial Diagnosis: Screening Colonoscopy, Diagnostic Colonoscopy, or Emergent Surgery, and Tumor Stage and Size at Initial Presentation. Clin Colorectal Cancer. 2016 Mar;15(1):67–73.

3. Diagnosis | Gastrointestinal tract (lower) cancers - recognition and referral | CKS | NICE [Internet]. [cited 2022 May 19]. Available from: https://cks.nice.org.uk/topics/gastrointestinal-tract-lower-cancers-recognition-referral/diagnosis/

4. Overview | Suspected cancer: recognition and referral | Guidance | NICE [Internet]. NICE; [cited 2022 Jan 21]. Available from: https://www.nice.org.uk/guidance/ng12

5. 1 Recommendations | Quantitative faecal immunochemical tests to guide referral for colorectal cancer in primary care | Guidance | NICE [Internet]. NICE; [cited 2022 Jan 21]. Available from: https://www.nice.org.uk/guidance/dg30/chapter/1-Recommendations

6. Hamilton W. The CAPER studies: five case-control studies aimed at identifying and quantifying the risk of cancer in symptomatic primary care patients. Br J Cancer. 2009 Dec 3;101 Suppl 2:S80-86.

7. Bailey SER, Abel GA, Atkins A, Byford R, Davies SJ, Mays J, et al. Diagnostic performance of a faecal immunochemical test for patients with low-risk symptoms of colorectal cancer in primary care: an evaluation in the South West of England. Br J Cancer. 2021 Mar;124(7):1231–6.

8. Pin-Vieito N, Tejido-Sandoval C, Vicente-Bielza N de, Sánchez-Gómez C, Cubiella J. Faecal immunochemical tests safely enhance rational use of resources during the assessment of suspected symptomatic colorectal cancer in primary care: systematic review and meta-analysis. Gut. 2022 May 1;71(5):950–60.

9. The NHS Bowel screening programme | Background information | Bowel screening | CKS | NICE [Internet]. [cited 2022 Feb 1]. Available from: https://cks.nice.org.uk/topics/bowel-screening/background-information/the-nhs-bowel-screening-programme/#bowel-cancer-screening-programme-in-england

10. Joint ACPGBI, BSG and BSGAR considerations for adapting the rapid access colorectoral cancer pathway during COVID-19 pandemic [Internet]. The British Society of Gastroenterology. 2020 [cited 2022 Jan 24]. Available from: https://www.bsg.org.uk/covid-19-advice/covid-19-advice-for-healthcare-professionals/joint-acgbbi-bsg-and-bsgar-considerations-for-adapting-the-rapid-access-colorectoral-cancer-pathway-during-covid-19-pandemic/

For peer review only

11. Maclean W, Limb C, Mackenzie P, Whyte MB, Benton SC, Rockall T, et al. Adoption of faecal immunochemical testing for 2-week-wait colorectal patients during the COVID-19 pandemic: an observational cohort study reporting a new service at a regional centre. Colorectal Dis. 2021;23(7):1622–9.

12. D'Souza N, Abulafi M. Navigating the storm of COVID-19 for patients with suspected bowel cancer. Br J Surg. 2020 May 13;107(7):e204.

13. Arasaradnam RP, Bhala N, Evans C, Greenaway J, Logan R, Penman I, et al. Faecal immunochemical testing in the COVID-19 era: balancing risk and costs. Lancet Gastroenterol Hepatol. 2020 Aug;5(8):717–9.

14. Faecal Immunochemical Testing (FIT) in patients with signs or symptoms of suspected colorectal cancer (CRC): A joint guideline from the Association of Coloproctology of Great Britain & Ireland (ACPGBI) and the British Society of Gastroenterology (BSG) [Internet]. The British Society of Gastroenterology. [cited 2022 Jun 10]. Available from: https://www.bsg.org.uk/clinical-resource/faecal-immunochemical-testing-fit-in-patients-with-signs-or-symptoms-of-suspected-colorectal-cancer-crc-a-joint-guideline-from-the-acpgbi-and-the-bsg/

15. Monahan KJ, Davies MM, Abulafi M, Banerjea A, Nicholson BD, Arasaradnam R, et al. Faecal immunochemical testing (FIT) in patients with signs or symptoms of suspected colorectal cancer (CRC): a joint guideline from the Association of Coloproctology of Great Britain and Ireland (ACPGBI) and the British Society of Gastroenterology (BSG). Gut [Internet]. 2022 Jul 12 [cited 2022 Aug 8]; Available from: http://gut.bmj.com/content/early/2022/07/25/gutjnl-2022-327985

16. Project information | Quantitative faecal immunochemical tests to guide colorectal cancer pathway referral for people with a change in bowel habit or abdominal pain | Guidance | NICE [Internet]. NICE; [cited 2022 Aug 8]. Available from: https://www.nice.org.uk/guidance/indevelopment/gid-dg10036

17. Chambers JA, Callander AS, Grangeret R, O'Carroll RE. Attitudes towards the Faecal Occult Blood Test (FOBT) versus the Faecal Immunochemical Test (FIT) for colorectal cancer screening: perceived ease of completion and disgust. BMC Cancer. 2016 Dec;16(1):1–7.

18. Bowyer HL, Vart G, Kralj-Hans I, Atkin W, Halloran SP, Seaman H, et al. Patient attitudes towards faecal immunochemical testing for haemoglobin as an alternative to colonoscopic surveillance of groups at increased risk of colorectal cancer. J Med Screen. 2013 Sep 1;20(3):149–56.

19. Chin YH, Ng CH, Seah SHY, Jain SR, Law JH, Devi MK, et al. Evolving perspectives on stool testing for colorectal cancer: a qualitative systematic review. Eur J Cancer Prev. 2020 Sep;29(5):416–23.

20. Johanna W, Yvonne W, Anna J, Kaisa F. Two sides of every coin: individuals' experiences of undergoing colorectal cancer screening by faecal immunochemical test and colonoscopy. Eur J Public Health. 2021 Dec 1;31(6):1290–5.

For peer review only

21. Vart G, Banzi R, Minozzi S. Comparing participation rates between immunochemical and guaiac faecal occult blood tests: a systematic review and meta-analysis. Prev Med. 2012 Aug;55(2):87–92.

22. Goodwin B, Viljoen B, Myers L, Ireland M, Dunn J, Chambers S, et al. Identifying modifiable features of home bowel cancer screening kits to facilitate use: consumer perspectives. Public Health Res Pract [Internet]. 2022 [cited 2022 Jun 27]; Available from: https://www.phrp.com.au/?p=41545

23. Delisle TG, D'Souza N, Davies B, Benton S, Chen M, Ward H, et al. Faecal immunochemical test for suspected colorectal cancer symptoms: patient survey of usability and acceptability. BJGP Open [Internet]. 2022 Jan 12 [cited 2022 Feb 8]; Available from: https://bjgpopen.org/content/early/2022/01/10/BJGPO.2021.0102

24. Calanzani N, Pannebakker M, Tagg MJ, Walford H, Holloway P, de Wit N,Hamilton W, Walter FM. Who are the patients being offered the faecal immunochemical test in routine English general practice, and for what symptoms? A prospective descriptive study. BMJ Open 2022. ((in press)).

25. Archer S, Calanzani N, Honey S, Johnson M, Neal R, Scott SE, et al. Impact of the COVID-19 pandemic on cancer assessment in primary care: a qualitative study of GP views. BJGP Open. 2021 Aug;5(4):BJGPO.2021.0056.

26. Scott SE, Walter FM, Webster A, Sutton S, Emery J. The Model of Pathways to Treatment: Conceptualization and integration with existing theory. Br J Health Psychol. 2013;18(1):45–65.

27. Walter F, Webster A, Scott S, Emery J. The Andersen Model of Total Patient Delay: a systematic review of its application in cancer diagnosis. J Health Serv Res Policy. 2012 Apr;17(2):110–8.

28. Sekhon M, Cartwright M, Francis JJ. Acceptability of health care interventions: A theoretical framework and proposed research agenda. Br J Health Psychol. 2018;23(3):519–31.

29. Forster AS, Rubin G, Emery JD, Thompson M, Sutton S, de Wit N, et al. Measuring patient experience of diagnostic care and acceptability of testing. Diagnosis. 2021 Aug 26;8(3):317–21.

30. Gale NK, Heath G, Cameron E, Rashid S, Redwood S. Using the framework method for the analysis of qualitative data in multi-disciplinary health research. BMC Med Res Methodol. 2013 Dec;13(1):117.

31. Best Qualitative Data Analysis Software for Researchers | NVivo [Internet]. [cited 2022 May 16]. Available from: https://www.qsrinternational.com/nvivo-qualitative-data-analysis-software/home

32. University of Cambridge | CanTest [Internet]. CanTest |. 2019 [cited 2022 Jun 7]. Available from: https://www.cantest.org/about/university-of-cambridge/

For peer review only - http://bmjopen.bmj.com/site/about/guidelines.xhtml

33. Regional ethnic diversity [Internet]. [cited 2022 Jun 23]. Available from: https://www.ethnicity-facts-figures.service.gov.uk/uk-population-by-ethnicity/national-and-regional-populations/regional-ethnic-diversity/latest

34. Vuik FE, Nieuwenburg SA, Bardou M, Lansdorp-Vogelaar I, Dinis-Ribeiro M, Bento MJ, et al. Increasing incidence of colorectal cancer in young adults in Europe over the last 25 years. Gut. 2019 Oct;68(10):1820–6.

35. Araghi M, Soerjomataram I, Bardot A, Ferlay J, Cabasag CJ, Morrison DS, et al. Changes in colorectal cancer incidence in seven high-income countries: a population-based study. Lancet Gastroenterol Hepatol. 2019 Jul 1;4(7):511–8.

36. Loomans-Kropp HA, Umar A. Increasing Incidence of Colorectal Cancer in Young Adults. J Cancer Epidemiol. 2019 Nov 11;2019:9841295.

37. Clarke N, Gallagher P, Kearney PM, McNamara D, Sharp L. Impact of gender on decisions to participate in faecal immunochemical test-based colorectal cancer screening: a qualitative study. Psychooncology. 2016;25(12):1456–62.

38. Litchfield IJ, Bentham LM, Lilford RJ, McManus RJ, Greenfield SM. Patient perspectives on test result communication in primary care: a qualitative study. Br J Gen Pract. 2015 Mar;65(632):e133–40.

39. Sampson R, Cooper J, Barbour R, Polson R, Wilson P. Patients' perspectives on the medical primary–secondary care interface: systematic review and synthesis of qualitative research. BMJ Open. 2015 Oct;5(10):e008708.

40. Banks J, Walter FM, Hall N, Mills K, Hamilton W, Turner KM. Decision making and referral from primary care for possible lung and colorectal cancer: a qualitative study of patients' experiences. Br J Gen Pract. 2014 Dec 1;64(629):e775–82.

41. Skivington K, Matthews L, Simpson SA, Craig P, Baird J, Blazeby JM, et al. A new framework for developing and evaluating complex interventions: update of Medical Research Council guidance. BMJ. 2021 Sep 30;374:n2061.

42. Accurx for Primary Care [Internet]. [cited 2022 Aug 8]. Available from: https://www.accurx.com/primary-care

For peer review only

For peer review only - http://bmjopen.bmj.com/site/about/guidelines.xhtml

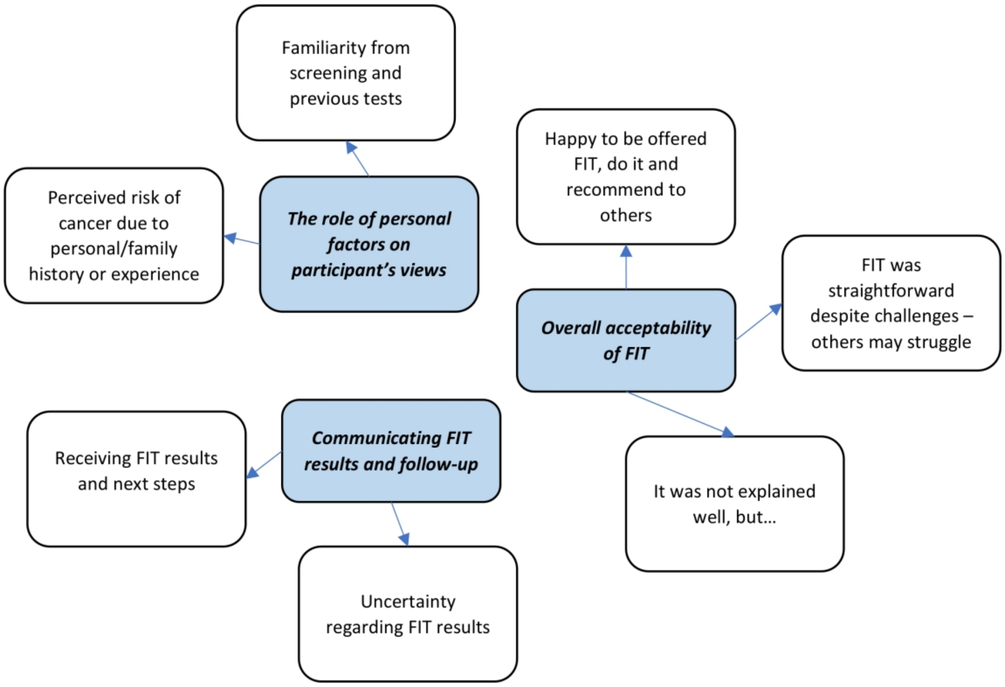

Figure 1. Themes and subthemes: patient experience and acceptability of using FIT

168x114mm (300 x 300 DPI)

Supplementary File 1. Interview topic schedule

Amended COVID items in red

Background

- Tell me a bit about you:
  - age
  - education
  - employment history
  - daily activities
  - general health

- What impact has coronavirus had on your everyday life?

Background to FIT

- What was your overall experience of the problem, getting tested and receiving your test result?

- Tell me more about the symptoms you experienced
  - Abdominal discomfort
  - Change in bowel habit (general, diarrhoea, constipation)
  - Bleeding
  - Indigestion / heartburn
  - Bloating
  - Fatigue
  - Unexplained weight loss
  - Loss of appetite.

- How long had you experienced symptoms?
  - Note: Try and get dates to be able to work out how this relates to COVID

- Did you try any self-management?
  - If yes, do you think coronavirus affected how you self-managed?

- When did you first seek help for symptoms?
  - Do you think coronavirus had any impact on when and where you sought help?
    - Friends and family
    - Other sources of information (e.g. internet)
    - Other medical professional (e.g. pharmacist)
  - Do you think coronavirus has changed the way you seek help for medical problems?
    - Changing thresholds for going to the doctors
    - Ongoing fear of going out
    - Change in the way that clinical appointments happen

- Did you take any prescription medication for symptoms?
  - How long taking
  - Any changes to medication and/or dose
  - How medication has helped
  - Aany problems?
  - Do you think coronavirus led to any problems getting medication?

Experience of GP consultation where the FIT-poo test was first suggested

- How they were feeling before GP appointment (about symptoms, urgency, what did they expect to happen)?

For peer review only

- Did coronavirus have any impact on how you interacted with your GP?
  - Did you have a face to face appointment?
  - Were there any remote part to the consultation?
  - Was this what you were expecting?

- What was it like to going to the GP practice during coronavirus?
  - How did this make you feel?
  - How do you feel about attending any future appointments with your GP?

- How did you feel when the GP suggested having a FIT-poo test?
  - How was the FIT-poo test introduced and explained?.
  - Who explained this (GP, nurse, admin?).
  - What did you think about the information you received (format and content).

- How did you feel following the GP consultation but before doing the test (physically and emotionally)?

- How long did you wait between the GP visit and doing the FIT-poo test at home?
  - What factors influenced this (busy/scared/anxious/collecting additional information?).
  - Did you discuss doing the FIT-poo test with anyone (family member, friend, GP, nurse)
  - What was your experience of discussion?
  - Did you look up or request any further information (including internet).
  - Any concerns about taking the test?

- Do you have any ideas about why people might decide against taking the test (why others might be put off)?

- What did you think the doctor was looking for?
  - Did you or they make any links between colorectal cancer and your these symptoms?

Experience of doing the FIT-poo test

- How did you feel about doing the test?
  - Physically
  - Emotionally
  - Did you manage first time you tried? If not, why not?

- How did you feel following the test?
  - Explore for immediately after test
  - Later that day
  - Following day
  - Over following week.
- Do you think the coronavirus made you more/less concerned about the test?

Experience of receiving the result of the FIT-poo test

- What was it like waiting for result?
  - Do you think coronavirus affected this?
  - For those who have not yet had a result – why do they think this might be?
  - How did you receive the test result (text message, letter, phone call, another consultation)
  - Do you think coronavirus has had any impact on the timeliness/delivery of results?

- What was it like being given a POSITIVE / NEGATIVE / EQIVOCAL result?

- Do you know what will happen next?
  - Are there any concerns about future care because of coronavirus?

For peer review only

- Do you think your experience has influenced any of your friends or family members about seeking/receiving care now and in the future?

Improvements to the FIT-poo test experience

- What went well before, during and after doing the FIT-poo test?
  - Any suggestions for changes that would improve FIT-poo test
    - Information provided
    - Procedure
    - Staff interpersonal skills
    - Experience of taking the FIT-poo test.

- What impact, if any, did coronavirus have on your testing experience?
  - Has this changed their view of future care and what this might look like post-covid?

- Would you take test again?

- Would you recommend test to someone else?
  - How would you explain test to someone else?

Ask whether anything else participant would like to mention / discuss

For peer review only

Supplementary File 2. Interviews in context: patients' experiences during the COVID-19 pandemic

Participants described feeling isolated from their support networks due to the limitations on social interactions during the pandemic. This resulted in some feeling unable to have conversations with family and friends regarding their symptoms during the appraisal period.

Many delayed seeking help as a result of what they perceived was happening within General Practice. A range of attitudes were displayed, from believing practices were overrun, to feeling GPs were 'shutdown' or only open for emergencies. For some, this resulted in the decision to seek help from an alternative provider such as 111 or walk-in services. In contrast, for a minority of participants positive media campaigns advising the public that the NHS remained open influenced them to contact their GP.

Changes to remote consulting (primarily in the form of telephone consultations) had both positive and negative impacts. Though many highlighted the convenience of telephone appointments and the speed of talking to their GP, others described the challenges of navigating complicated booking systems, and describing problems over the phone.

For those that visited their GP surgery during the pandemic, personal protective equipment and social distancing measures were generally expected and accepted, despite often feeling time-consuming or frustrating. Many perceived GP practices to be safer and cleaner than other public spaces.

For peer review only

COREQ (COnsolidated criteria for REporting Qualitative research) Checklist

A checklist of items that should be included in reports of qualitative research. You must report the page number in your manuscript where you consider each of the items listed in this checklist. If you have not included this information, either revise your manuscript accordingly before submitting or note N/A.

Topic	Item No.	Guide Questions/Description	Reported on Page No.
**Domain 1: Research team and reflexivity**			
Personal characteristics			
Interviewer/facilitator	1	Which author/s conducted the interview or focus group?	
Credentials	2	What were the researcher's credentials? E.g. PhD, MD	
Occupation	3	What was their occupation at the time of the study?	
Gender	4	Was the researcher male or female?	
Experience and training	5	What experience or training did the researcher have?	
Relationship with participants			
Relationship established	6	Was a relationship established prior to study commencement?	
Participant knowledge of the interviewer	7	What did the participants know about the researcher? e.g. personal goals, reasons for doing the research	
Interviewer characteristics	8	What characteristics were reported about the inter viewer/facilitator? e.g. Bias, assumptions, reasons and interests in the research topic	
**Domain 2: Study design**			
Theoretical framework			
Methodological orientation and Theory	9	What methodological orientation was stated to underpin the study? e.g. grounded theory, discourse analysis, ethnography, phenomenology, content analysis	
Participant selection			
Sampling	10	How were participants selected? e.g. purposive, convenience, consecutive, snowball	
Method of approach	11	How were participants approached? e.g. face-to-face, telephone, mail, email	
Sample size	12	How many participants were in the study?	
Non-participation	13	How many people refused to participate or dropped out? Reasons?	
Setting			
Setting of data collection	14	Where was the data collected? e.g. home, clinic, workplace	
Presence of non-participants	15	Was anyone else present besides the participants and researchers?	
Description of sample	16	What are the important characteristics of the sample? e.g. demographic data, date	
Data collection			
Interview guide	17	Were questions, prompts, guides provided by the authors? Was it pilot tested?	
Repeat interviews	18	Were repeat inter views carried out? If yes, how many?	
Audio/visual recording	19	Did the research use audio or visual recording to collect the data?	
Field notes	20	Were field notes made during and/or after the inter view or focus group?	
Duration	21	What was the duration of the inter views or focus group?	
Data saturation	22	Was data saturation discussed?	
Transcripts returned	23	Were transcripts returned to participants for comment and/or	

For peer review only

Topic	Item No.	Guide Questions/Description	Reported on Page No.
		correction?	
**Domain 3: analysis and findings**			
Data analysis			
Number of data coders	24	How many data coders coded the data?	
Description of the coding tree	25	Did authors provide a description of the coding tree?	
Derivation of themes	26	Were themes identified in advance or derived from the data?	
Software	27	What software, if applicable, was used to manage the data?	
Participant checking	28	Did participants provide feedback on the findings?	
Reporting			
Quotations presented	29	Were participant quotations presented to illustrate the themes/findings? Was each quotation identified? e.g. participant number	
Data and findings consistent	30	Was there consistency between the data presented and the findings?	
Clarity of major themes	31	Were major themes clearly presented in the findings?	
Clarity of minor themes	32	Is there a description of diverse cases or discussion of minor themes?	

Developed from: Tong A, Sainsbury P, Craig J. Consolidated criteria for reporting qualitative research (COREQ): a 32-item checklist for interviews and focus groups. *International Journal for Quality in Health Care*. 2007. Volume 19, Number 6: pp. 349 – 357

Once you have completed this checklist, please save a copy and upload it as part of your submission. DO NOT include this checklist as part of the main manuscript document. It must be uploaded as a separate file.

For peer review only

BMJ Open

Can we do better? A qualitative study in the East of England investigating patient experience and acceptability of using the faecal immunochemical test in primary care

Journal:	*BMJ Open*
Manuscript ID	bmjopen-2023-072359.R1
Article Type:	Original research
Date Submitted by the Author:	24-Apr-2023

Complete List of Authors:	Snudden, Claudia; University of Cambridge Department of Public Health and Primary Care,<br>Calanzani, Natalia; University of Aberdeen Institute of Applied Health Sciences, Academic Primary Care; University of Cambridge Department of Public Health and Primary Care<br>Archer, Stephanie; University of Cambridge Department of Public Health and Primary Care<br>Honey, Stephanie; University of Leeds Leeds Institute of Health Sciences<br>Pannebakker, Merel; University of Cambridge Department of Public Health and Primary Care<br>Faher, Anissa; University of Cambridge School of Clinical Medicine<br>Chang, Aina; University of Cambridge School of Clinical Medicine<br>Hamilton, Willie; University of Exeter<br>Walter, Fiona M; Queen Mary University of London, Wolfson Institute of Population Health; University of Cambridge, Department of Public Health and Primary Care

**Primary Subject Heading**:	General practice / Family practice
Secondary Subject Heading:	Oncology
Keywords:	PRIMARY CARE, QUALITATIVE RESEARCH, GASTROENTEROLOGY

SCHOLARONE™
Manuscripts

I, the Submitting Author has the right to grant and does grant on behalf of all authors of the Work (as defined in the below author licence), an exclusive licence and/or a non-exclusive licence for contributions from authors who are: i) UK Crown employees; ii) where BMJ has agreed a CC-BY licence shall apply, and/or iii) in accordance with the terms applicable for US Federal Government officers or employees acting as part of their official duties; on a worldwide, perpetual, irrevocable, royalty-free basis to BMJ Publishing Group Ltd ("BMJ") its licensees and where the relevant Journal is co-owned by BMJ to the co-owners of the Journal, to publish the Work in this journal and any other BMJ products and to exploit all rights, as set out in our licence.

The Submitting Author accepts and understands that any supply made under these terms is made by BMJ to the Submitting Author unless you are acting as an employee on behalf of your employer or a postgraduate student of an affiliated institution which is paying any applicable article publishing charge ("APC") for Open Access articles. Where the Submitting Author wishes to make the Work available on an Open Access basis (and intends to pay the relevant APC), the terms of reuse of such Open Access shall be governed by a Creative Commons licence – details of these licences and which Creative Commons licence will apply to this Work are set out in our licence referred to above.

Other than as permitted in any relevant BMJ Author's Self Archiving Policies, I confirm this Work has not been accepted for publication elsewhere, is not being considered for publication elsewhere and does not duplicate material already published. I confirm all authors consent to publication of this Work and authorise the granting of this licence.

For peer review only

Can we do better? A qualitative study in the East of England investigating patient experience and acceptability of using the faecal immunochemical test in primary care

Claudia M Snudden[1] BMBCh (ORCID ID: 0000-0002-7355-4317)
Natalia Calanzani[1,2] PhD (ORCID ID: 0000-0002-5068-2543)
Stephanie Archer[1] PhD (ORCID ID: 0000-0003-1349-7178)
Stephanie Honey[3] PhD (ORCID ID: 0000-0002-2675-7824)
Merel M Pannebakker[1] PhD (ORCID ID: 0000-0003-2918-0570)
Anissa Faher[4] BA Hons (Cantab)
Aina Chang[4] BA Hons (Cantab)
Willie Hamilton[5] FRCGP (ORCID ID: 0000-0003-1611-1373)
Fiona M Walter[6,1] FRCGP (ORCID ID: 0000-0002-7191-6476)

[1] The Primary Care Unit, Department of Public Health and Primary Care, University of Cambridge, Cambridge, UK
[2] Academic Primary Care, Institute of Applied Health Sciences, University of Aberdeen, Aberdeen, UK
[3] Leeds Institute of Health Sciences, University of Leeds, Leeds, UK
[4] School of Clinical Medicine, Addenbrooke's Hospital, University of Cambridge, UK
[5] University of Exeter Medical School, Exeter, UK
[6] Wolfson Institute of Population Health, Queen Mary University of London, UK

Corresponding author: Dr Claudia Snudden
Email: cs2048@medschl.cam.ac.uk

Address: The Primary Care Unit, Department of Public Health and Primary Care, University of Cambridge, Cambridge, UK

Word count: 4417

For peer review only

ABSTRACT

Objectives

The faecal immunochemical test (FIT) is increasingly used in UK primary care to triage patients presenting with symptoms and at different levels of colorectal cancer risk. Evidence is scarce on patients' views of using FIT in this context. We aimed to explore patients' care experience and acceptability of using FIT in primary care.

Design

A qualitative semi-structured interview study. Interviews were conducted via Zoom between April and October 2020. Transcribed recordings were analysed using framework analysis.

Setting

East of England General Practices.

Participants

Consenting patients (aged ≥40 years) who presented in primary care with possible symptoms of colorectal cancer, and for whom a FIT was requested, were recruited to the FIT-East study. Participants were purposively sampled for this qualitative sub-study based on age, gender, and FIT result.

Results

44 participants were interviewed with a mean age 61 years, and 25 (57%) being male: 8 (18%) received a positive FIT result. Three themes and seven subthemes were identified. Participants' familiarity with similar tests and perceived risk of cancer influenced test experience and acceptability. All participants were happy to do the FIT themselves and to recommend it to others. Most participants reported that the test was straightforward, although some considered it may be a challenge to others. However, test explanation by healthcare professionals was often limited. Furthermore, while some participants received their results quickly, many did not receive them at all with the common assumption that "no news is good news". For those with a negative result and persisting symptoms, there was uncertainty about any next steps.

For peer review only

For peer review only - http://bmjopen.bmj.com/site/about/guidelines.xhtml

Conclusions

While FIT is acceptable to patients, elements of communication with patients by the healthcare system show potential for improvement. We suggest possible ways to improve the FIT experience, particularly regarding communication about the test and its results.

For peer review only

For peer review only - http://bmjopen.bmj.com/site/about/guidelines.xhtml

STRENGTHS AND LIMITATIONS OF THIS STUDY

- Interested participants were contacted within four weeks of performing FIT, helping to reduce recall bias
- All participants were White (British or other) and English speakers – views may differ for those identifying with other ethnicities or who do not understand English well
- The use of Zoom for participant interviews may have hindered those unable to navigate the required technology from taking part

For peer review only

INTRODUCTION

Colorectal cancer (CRC) is the third most common cancer worldwide and incidence is increasing[1]. Globally, mortality rates are decreasing, in part due to increased screening, improved early diagnosis, and advances in treatment[2,3]. Within the United Kingdom (UK), the majority of patients diagnosed with CRC will first present to their General Practitioner (GP) with abdominal symptoms[4,5]. GPs act as gatekeepers within the UK's National Health Service (NHS), deciding which patients require onwards referrals to secondary care, and how urgently.

Diagnosing CRC, particularly in its early stages, can be difficult as symptoms of CRC are broad and non-specific[6]. GPs in England currently follow National Institute for Health and Care Excellence (NICE) 2015 guidelines for patients with suspected cancer to identify higher risk patients who should be urgently referred for further investigation[7]. In 2017, NICE recommended the use of the Faecal Immunochemical Test (FIT) within primary care to help triage low risk patients with possible CRC symptoms that do not meet urgent referral criteria[2,8]. FIT can be used for both ruling in and ruling out CRC[9,10]. Alongside its use in symptomatic patients, FIT is also used in the UK's bowel cancer screening programme, having replaced the previously used guaiac faecal occult blood test (gFOBT)[11].

The threshold set to define a positive FIT result (i.e., the haemoglobin cut-off concentration) differs between populations. For symptomatic patients, NICE advises that those with a FIT result ≥ 10μg Hb/g faeces are referred on for further diagnostic testing; of these about 7% will be diagnosed with CRC[2,9]. In contrast, England uses a cut-off of 120μg Hb/g in asymptomatic screening to select who requires further investigation[11]. Variations in thresholds are due to the differences in risk between asymptomatic and symptomatic populations; the risk of cancer is higher for those with symptoms therefore the threshold for further investigations is lower.

As a result of the COVID-19 pandemic, FIT use in primary care was intensified to help prioritise urgent referrals[12–15]. In June 2022, The British Society of Gastroenterology released additional guidelines recommending the continued use of FIT as a diagnostic triage tool for all urgent referrals[16,17]. NICE also plans to publish additional guidance on FIT by November 2023[18]. Given the increasing reliance of FIT use in primary care, it is critical to understand what patients think of the test and whether they find it acceptable.

Studies conducted in screening contexts have shown that disgust associated with performing stool tests and certain procedural aspects of FIT can present barriers to uptake, however, the

For peer review only - http://bmjopen.bmj.com/site/about/guidelines.xhtml

test is often found acceptable[19–24]. Nonetheless, there is still limited research into FIT experience and acceptability, particularly within the context of symptomatic patients[25]. Understanding both care experience and acceptability is vital to ensure good quality of care for patients[26].

Since symptomatic populations have varied (and higher) levels of risk compared to an asymptomatic screening population, evidence on the patients' experience of care when and after doing FIT is crucial. To our knowledge, only two other studies have reported on symptomatic patients' views of FIT; one quantitative study focusing on usability and acceptability[25], and one mixed-methods study evaluating patient experience and satisfaction[27].

Therefore, this study aims to build on existing literature, providing an in-depth qualitative exploration of both FIT experience and acceptability for patients presenting to primary care with possible symptoms of CRC.

For peer review only - http://bmjopen.bmj.com/site/about/guidelines.xhtml

METHODS

Design, setting and population

This qualitative sub-study was undertaken as part of the FIT-East study, which was set across General Practices in the East of England Cancer Alliance (Suffolk & North East Essex and Norfolk & Waveney)[28]. FIT-East included 507 patients aged 40 years and older with possible symptoms of CRC, but who did not immediately meet urgent referral criteria, for whom a FIT was requested by the GP[28]. FIT kits contained a study recruitment letter and consent form; participants could tick a box if interested in taking part in interviews.

We undertook semi-structured interviews with patients to investigate experience and acceptability of using FIT in primary care. Interviews were also carried out with General Practitioners (reported elsewhere[29]). We followed the Consolidated Criteria for Reporting Qualitative studies (COREQ) guidelines[30]. Ethical approval was granted by the East of England Cambridge Central Research Ethics Committee – reference 19/EE/0036.

Sampling

We undertook purposive sampling based on age, gender, and FIT result to obtain a range of experiences among patients who consented to take part in the FIT-East study. Potential participants were contacted by email (provided by patients in the consent forms). To reduce recall bias, we contacted patients within four weeks of FIT being sent to the laboratory.

Ninety-nine participants responded positively to the initial email and were invited for interview. Additional informed consent was required and obtained for all patients. Interviews were carried out until no new topics were discussed: 45 participants were interviewed, mean duration 39.5 minutes. Due to the COVID-19 pandemic, interviews were carried out remotely using the Zoom platform, and audio-recorded. One interview could not be analysed due to a technical fault during recording; therefore 44 participants were included. Each participant was individually interviewed once by one or two experienced female health services researchers with a background in Psychology (SA and MMP) or Nursing (SH).

Interview topic guide

The guide (Supplementary File 1) explored patient experiences of being asked to undertake a FIT in primary care, covering areas including: obtaining the test, using it, returning it, and

For peer review only - http://bmjopen.bmj.com/site/about/guidelines.xhtml

receiving the results. The topic guide was piloted by the research team before use. In order to better understand patients' experiences during the COVID-19 pandemic, additional questions were added in June 2020. Interviews were carried out between April and October 2020.

Data analysis

Data analysis was informed by three conceptual models: the Model of Pathways to Treatment[31,32], the Theoretical Framework of Acceptability (TFA)[33] and Forster's *et al* considerations when assessing acceptability of diagnostic tests[26]. We chose these three models to underpin important complementary but distinctive aspects of the cancer diagnostic pathway[31,32] including the role of patient factors/characteristics[31,32], the construct of acceptability[33] and patient's experience of care[26]. Acceptability is defined as a multi-faceted construct comprising affective attitude, burden, perceived effectiveness, ethicality, intervention coherence, opportunity costs, and self-efficacy[33].

Verbatim transcripts were checked, anonymised and analysed using framework analysis[34]. Four researchers (one academic GP [CS], one health services researcher [NC], and two medical students [AF and AC]) repeatedly read the transcripts and coded the interviews. Eight interviews were initially coded inductively by CS to discover potentially unexpected aspects of the participants' experiences. An analytical framework was then developed using codes from the eight initial transcripts; codes were grouped into categories, influenced by the relevant aspects of the three models. The framework was then used to systematically index the remaining transcripts, including double coding of a quarter of the data set. Codes and categories were constantly refined during analysis. Data were charted into a series of matrices from which themes were developed. Consistency of coding was discussed regularly during team meetings; themes were also amended with guidance from senior team members (SA and FMW). NVivo 12 was used to facilitate analysis[35].

Participant age (range), gender (M for male and F for female) and FIT results (positive, negative or unclear) are reported alongside quotes to aid interpretation of findings. Quotes were chosen to illustrate findings while also ensuring a good spread of participants and their characteristics.

Patient and public involvement

Patient-public partners from the CanTest Collaborative[36] (responsible for FIT-East) provided feedback on the original and amended interview topic guides. Representatives also read and

For peer review only

commented on interview transcripts; these comments were considered when iteratively revising our analysis.

For peer review only

RESULTS

Table 1 describes characteristics of the included participants and describes how FIT results were received. About a fifth (18%) had a positive FIT result. Outcome data for whether participants received a cancer diagnosis was not available.

Table 1. Demographic characteristics and FIT results (n=44)

Characteristic	Variable	N	%
Sex	Female	19	43
	Male	25	57
Age (years)	40-49	3	7
	50-59	17	39
	60-69	10	23
	70-79	11	25
	80-89	3	7
FIT result	Negative	35	80
	Positive	8	18
	Unclear*	1	2
How FIT result was received	Patient called practice†	12	27
	Patient did not receive results/is unsure‡	11	25
	Practice called patient§	9	20
	Patient told face-to-face‖	4	9
	Patient received letter¶	3	7
	Patient checked results online¶	2	5
	Other**	3	7
Education level	None	3	7
	GCSE (or equivalent)	4	9
	A level (or equivalent)	5	11
	Degree (or equivalent)	18	41
	Other higher education	10	23
	Missing	4	9
Ethnicity	White British	35	80
	White Irish	1	2
	Any other White background	2	4
	Missing	6	14
Indices of Multiple Deprivation (IMD) quintiles	1 (most deprived)	0	0
	2	4	9
	3	7	16
	4	4	9
	5 (least deprived)	6	14
	Missing	23	52

For peer review only - http://bmjopen.bmj.com/site/about/guidelines.xhtml

	* One patient described doing the FIT in detail, but on the patient database FIT was recorded as "not done". As the patient clearly had done the test, this was recorded as an unclear result.<br>†One positive result (patient reported this as inconclusive during the interview).<br>‡Did not receive (n=7, all negative), unsure about it (n=4, 2 negative, one positive and one missing)<br>§GP (n=6, 2 positive results), nurse practitioner (n=1, negative – nurse called about something else and the patient asked for results); receptionist (n=2, 1 positive result).<br>‖GP (n=2, all negative), nurse (n=1, positive), receptionist (n=1, negative)<br>¶ All negative results<br>**Cannot remember how results were received (n=1, positive), GP contacted but unclear if by phone or face-to-face (n=1, negative), over the phone but unclear who called whom (n=1, positive).

Overview and Context

We identified three themes and seven subthemes describing patient experience and acceptability of using FIT after presenting in primary care with possible symptoms of CRC (but not meeting immediately criteria for urgent referral) (Figure 1).

As the study was performed during the COVID-19 pandemic a brief description of COVID-19 related themes is available for context (Supplementary file 2). The pandemic did not appear to influence participants' experiences of using FIT, but it did affect how participants accessed their GP prior to receiving the test.

The role of personal factors on participant's views

Perceived risk of cancer due to personal/family history or experience

A personal history of having had cancer in the past encouraged participants to seek help quickly for their symptoms. Similarly, cancer experiences from family and friends influenced their decision to seek help after noticing body changes, and to do the test when it was offered:

> *"My father has had bowel cancer. He basically had it once and they took a section of the colon out. After a few years it returned so they removed the whole of his colon and he's now been…you know, God bless he's still alive and it's been sort of over 15 years he's been clear but there is that sort of history that I was aware of. So I thought, alright, better…you know, because it had changed slightly, yes, and it was bleeding more often, I thought I'd better go and get it looked at."* (PT18, M, 50-59y, negative)

Participants became concerned that their symptoms could be a sign of bowel cancer - either from their own knowledge of 'red flag' symptoms or from reading about symptoms online, leading them to actively seek advice. Others became increasingly anxious or concerned about

For peer review only - http://bmjopen.bmj.com/site/about/guidelines.xhtml

the severity of their symptoms, particularly if things had worsened over time or were causing significant discomfort.

On the other hand, having had a relatively recent examination or negative investigation (such as colonoscopy) often reassured them there was nothing serious and being offered a FIT was not considered to be worrying or concerning.

Familiarity from screening and previous tests

Participants were familiar with doing stool tests, usually as part of bowel screening or previous diagnostic processes. As a result, they often displayed no concerns with the idea of doing FIT:

> *"Well, I've been doing them for years. It didn't upset me, if that's what you mean. It's just one more thing, going to the bog isn't it? Going to the loo? Except this time you put it on a piece of […] paper, and get a smear and stick it in the packet, and then seal it up."* (PT1, M, 70-79y, negative)

Participants who had had previous experiences within secondary care (such as colonoscopies due to diverticular disease) described the FIT experience as less daunting:

> *"I was quite relaxed about* [being asked to do FIT] *because I'd had a colonoscopy before, that was five years ago, so that was more nerve-racking."* (PT25, F, 60-69y, negative)

Overall acceptability of FIT

Happy to be offered FIT, do it and recommend it to others

Overall, participants reported they were glad or relieved to be offered FIT and accepted the need to do it. This was true even when the potential unpleasantness or difficulties of doing a FIT were recognised. For some, doing a test could lead to an 'earlier diagnosis' and the potential to start treatment sooner if there was something serious going on. For others, acceptance was associated with wanting to find an answer to their symptoms, especially if these had been particularly distressing:

For peer review only - http://bmjopen.bmj.com/site/about/guidelines.xhtml

> *"I guess I felt a little bit strange, kind of, like here I am, you know, lining the toilet to catch my stools. But when you know that it's something that you want to get done and want to get answers for you just get on and do it really."* (PT12, F, 40-49y, negative)

When reflecting on why *others* might not want to do FIT, common reasons included the fear of a potential cancer diagnosis and finding the process disgusting or embarrassing:

> *"Probably because it's not a very nice thing to have to do, you know. Playing around with poo is not something that everybody thinks, 'oh that sounds like fun'."* (PT3, F, 60-69y, negative)

Every participant reported being happy to repeat FIT if necessary, and all would willingly recommend FIT to others if they needed it.

FIT was straightforward despite challenges – others may struggle

Participants commented on challenges they faced when physically collecting the FIT sample: often these were difficulties writing the label, 'catching' the stool sample without it falling into the toilet water or when suffering with diarrhoea, and finding the FIT stick small or *"very fiddly"* to handle.

Anxiety was also expressed regarding taking the FIT sample and "*wanting to do it right*". Furthermore, participants reported wanting to get the sample done and feeling relieved after completing the test:

> *"I wanted to get it out of the way, it's not exactly difficult, but it was just the having to, you know, deal with it. So I woke up thinking, 'oh, here we go, you know, I need to do this' and when I'd got things in the specimen jars and I sorted myself out, I was glad it was over."* (PT23, F, 50-59y, negative)

Methods to overcome difficulties collecting the sample were also described, drawing on prior experiences when completing bowel cancer screening tests. Nonetheless, participants (even those reporting these challenges) were confident about their ability to do the test, often remarking how simple and 'straightforward' FIT was.

For peer review only - http://bmjopen.bmj.com/site/about/guidelines.xhtml

In particular, participants found the instructions generally easy to follow. Nonetheless, they discussed why *others* might struggle with FIT – from potential difficulties faced by having arthritic or shaky hands, poor eyesight, or an upset stomach, to issues following instructions.

It was not explained well, but…

Participants agreed that the explanations given by their healthcare professional (usually a GP or practice nurse) regarding what FIT was and why it was indicated, tended to be poor:

> *"My GP didn't, actually, tell me what it was. I had to Google it to even find out what it was. He said 'we'll leave you out a FIT test', and I had no idea what it was."* (PT14, M, 60-69y, positive)

Nonetheless, some defended the professionals in such situations, attributing the limited information to common constraints in a consultation such as limited time to go through different medical problems.

Often participants were asked to do multiple stool tests as part of their symptom work-up, leading to confusion regarding the differences between tests. Frequent references were made to being handed a bag containing a FIT and being told to just follow the instructions. For two participants, the poor communication even resulted in confusion of the test with the similarly named Fitbit watch.

Whilst the text explanation was often inadequate, participants had very good intuition about linking FIT to CRC. Participants reported that GPs alluded to cancer but did not use the word 'cancer' specifically, choosing instead to describe it as "*something sinister*" or "*something nasty lurking*". Despite this, many appeared to interpret the GP's language appropriately:

> *"When he suggested doing a FIT test, which he explained to me what it was, I thought 'yeah, it makes sense really because I know what you're after'. He didn't tell me what he was after, but I thought 'I know what you're looking for'."* (PT24, M, 50-59y, negative)

Participants' understanding of how FIT worked varied considerably. The most common explanations focused around looking for traces of (often non-visible) blood in the stool, but others described FIT as "*a bit of an MOT check*", being used to "*determine the health of the*

For peer review only - http://bmjopen.bmj.com/site/about/guidelines.xhtml

gut", "*check the digestive system is working properly*" or look for "*abnormalities within the poo*". The internet was sometimes used to further research FIT and causes of blood in the stool.

Whilst most participants demonstrated good intuition or a basic understanding of FIT, some had no awareness as to why they were asked to do FIT, yet did it anyway:

> *"I just got given the test to do and, I suppose, in a roundabout, sort of, way, she was saying, you know, 'you need to have the test done', but I didn't understand it and I was puzzled about it [...] I think that's probably why I kept reading the notes about it, because I kept thinking, 'well, I'm not sure why I'm doing this'."* (PT33, F, 50-59y, negative)

Participants also talked about trusting the GP and their advice, and how this influenced their decision to do the FIT, how it would affect their decision to do it again or recommend it to others:

> *"I like her, I think she's an excellent GP. If she asks me to do something, to participate or provide a test for example, I trust her to be asking that for a good reason and I will instinctively comply with any requests for tests or anything else as I'm assuming that it's going to provide information, test results or whatever which will be for my direct benefit."* (PT19, M, 60-69y, negative)

Communicating FIT results and follow-up

Uncertainty regarding FIT results

Participants reported contrasting attitudes while waiting for test results. Some discussed the anxiety surrounding the waiting period, at times hypothesising what would happen next in the worst-case scenario.

In contrast, others were unaffected by waiting, seemingly managing to *"forget about it"* and carry on with life as usual. This was often linked to either normalising FIT to a more 'standard' test "*just like having a blood test*", or expecting to receive a negative result and therefore not seeing reason to worry.

Participants felt they weren't given clear information by the GP on when and how to access their results. They suggested that chasing results should be practice-led rather than patient-led,

For peer review only - http://bmjopen.bmj.com/site/about/guidelines.xhtml

regardless of the result. Some participants did not receive their results at all, others contacted the practice for these, and others assumed that the practice would only contact them if there was a positive result and reported not being worried as *"no news is good news"*:

> *"I haven't actually been told anything so I presume they were negative or there were no concerns because no GP has contacted me."* (PT20, F, 50-59y, negative)

Receiving FIT results and next steps

For those who received their test results, there were often comments on receiving these quickly. Nonetheless, the way results were communicated was not always well received, both for positive and negative results:

> *"The receptionist at the GP surgery telephoned me and said the doctor had referred me[...] and I said, 'oh, why is that then', and she...she didn't seem to know and I said 'I just had a FIT test, what was the result of that'? Pause, and she said, 'oh, yes, it was positive. I expect that's why it is'. And I'm not joking, that's exactly how the conversation went.*" (PT14, M, 60-69y, positive)

> *"I think one slightly disappointing aspect I suppose is that when I phoned for results, I only spoke to the receptionist, and all they were able to tell me was no further action necessary. And I kind of thought, 'oh well, it's great in one way and I was pleased with that, but it kind of didn't explain why I'd had problems earlier' and I thought, 'oh, do I just wait to see if I ever have this problem again'."* (PT41, M, 50-59y, negative)

For participants with a positive result, there were reported delays in having a colonoscopy, particularly during the COVID-19 pandemic. Participants were often stoical about this situation. For those with a negative result, a sense of relief and reassurance was frequently reported. However, when symptoms persisted, there was uncertainty regarding what to do next. Some said they would have valued a follow up consultation with their GP, but in practice this rarely happened, leaving participants feeling their problems had not been fully resolved. Others believed that the GP would not be able to offer them anything further even if they did arrange a further appointment:

For peer review only - http://bmjopen.bmj.com/site/about/guidelines.xhtml

“No, I don’t know if there’s a follow-up or anything, if you detect nothing does it just get swept under the carpet or do I stay on your database and do you check up again or…I don’t know?” (PT6, M, 60-69y, negative)

For peer review only

For peer review only - http://bmjopen.bmj.com/site/about/guidelines.xhtml

DISCUSSION

Summary

This study of patients presenting in primary care with symptoms of possible CRC but not immediately meeting criteria for urgent referral found FIT an acceptable and straightforward test, despite some challenges manipulating the test kit. Participant characteristics such as past test experiences and perceived personal risk of cancer influenced attitudes towards FIT. Participants were also happy to do FIT again and recommend it to others, and trusted that their GP knew what was best for them. On the other hand, test explanations from health professionals were generally poor. This could be due, in part, to the lack of clarity surrounding FIT implementation and diagnostic pathways within primary care during the COVID-19 pandemic. There was also reported dissatisfaction with the way results were received, for both positive and negative results. Many participants did not receive results at all, and several assumed that "*no news is good news*". For some, there was no follow-up after a negative result, even when symptoms persisted.

Strengths and limitations

To our knowledge, this is the first qualitative study to explore experience and acceptability of FIT for symptomatic patients. We provide rich data on patients' care experiences and acceptability of FIT use in primary care. With the increased use of FIT in this setting, it is essential to understand patient views on the test and identify areas where care needs to be improved. Three well-established theoretical models were used to guide the analysis[26,31–33]. We found that the models complemented each other, with overlap between different elements. Patient factors[31] such as perception of risk, previous knowledge and experience influenced test acceptability[33], including the perceived burden, understanding why the test was being done (intervention coherence) and perceived ability to do the test (self-efficacy). Conversely, the overall test acceptability contrasted with the often reported less than ideal experience of care[26] before, during and after doing the test. Using a single model alone would have resulted in missing several nuances regarding acceptability, the role of patient factors and the quality of the experienced care.

Interested participants were contacted within four weeks of performing FIT, helping to reduce recall bias. There was good variation amongst participants in terms of age, gender and FIT result. However, only a sample of patients who took part in the FIT-East study were interviewed;

For peer review only - http://bmjopen.bmj.com/site/about/guidelines.xhtml

views of those who did not participate in the interviews may differ. The sample was also limited to the East of England. All participants identified themselves as White, slightly higher than the proportion reported by the UK Census for this region[37], and understood English well. Experiences of patients identifying with other ethnicities, and of those who do not speak English well may differ and need to be further investigated. Limited evidence on FIT use in symptomatic patients indicates that ethnicity may influence patient willingness to do the test[25]. Finally, we were not able to explore variations in views based on different levels of socioeconomic deprivation due to large amounts of missing data (52%). The relationship between poor care experience and test acceptability may differ for these groups; different levels of knowledge and experience may also play a role on FIT acceptability.

Due to resulting restrictions from the pandemic, interviews were conducted over Zoom, hindering those unable to navigate the required technology from taking part., No patients aged under 40 were recruited (as defined by our inclusion criteria). As FIT becomes more widely used in primary care, it will be key to explore the attitudes of this group, less familiar with stool testing and without involvement in prior screening programmes – particularly as the incidence of CRC is increasing in the under 50s[38–40].

Comparison with existing literature

Consistent with existing literature (although mostly investigating asymptomatic populations), FIT is seen as relatively straightforward with clear instructions[21–23,25]. Nonetheless, similar challenges to collecting the sample were reported including difficulties writing the label and handling the FIT stick[22,24]. Familiarity with stool tests is associated with higher intentions to use related tests in future, aligning with the confidence displayed by participants with experience of screening[25]. Social influences have also been associated with encouragement of FIT uptake, impacting an individual's perceived risk of, or susceptibility to, cancer[21,41].

Participants displayed different levels of proactivity in seeking FIT results, reflective of behaviours seen in the literature[42]. Patients reported wanting further clarification on how results are communicated; existing uncertainties were anxiety-inducing at times[22,42,43]. To our knowledge, there is no other available evidence on follow-up (or lack thereof) of patients with persisting symptoms and a negative FIT result.

Implications for research, practice and policy

For peer review only

Building on our findings and published literature, we have developed recommendations for research, practice and policy to improve both the FIT experience and patient safety (Box 1). Rationale for these recommendations is described below.

While FIT acceptance was high, there are still opportunities for improvement, particularly regarding processes to collect the stool sample, and better understanding FIT acceptability across different population groups.

Reports of questionable care about explaining the test, providing results and following up are also concerning[44]. While participants still found FIT acceptable and trusted that the GP knew what was good for them, care experiences caused anxiety to patients and indicate risks regarding continuity of care. Time waiting for results did not seem to be a problem for those who got them; but many reported that they never received the results. While no news was indeed often good news, some patients who chased their results found out they were positive. It is vital that GPs are clearer about when results are expected and how to access them, and safety-netting measures are in place to avoid missing cancers and provide better support to patients.

Finally, our results can support policy makers regarding FIT use and implementation within primary care. While flexibility and adaptability are required in the provision of services so different needs can be met[45], it is vital that national policies define standards on acceptable levels of service, particularly regarding how and when information on test results are provided.

Box 1. Recommendations for practice, research and policy

Research

- Investigate 1) challenges in collecting the stool sample, particularly for older people and those with mobility issues; and 2) improvements to sample collection (such as providing a collection container) and instructions (such as clarification on what to do in the case of diarrhoea).
- Investigate whether patients' presenting symptoms (high vs low risk) influence their perception of FIT, as we found that perceived risk and susceptibility influenced acceptability
- Seek to understand the views of patients who choose not to complete FIT after being offered it, to determine 1) why FIT may not be acceptable to some and 2) whether reasons align with issues identified in this study (e.g., test burden and limited information).
- Investigate the views of those living in areas of higher deprivation, and those identifying with different ethnic groups other than White.

Practice

For peer review only - http://bmjopen.bmj.com/site/about/guidelines.xhtml

- Work to improve GP communication with patients regarding why FIT is being done, what it looks for, and what a positive result means. Clear communication can also concurrently reduce patient confusion when multiple tests are requested simultaneously.
- Consider implementing a specific time slot for calling for results and promote these changes to patients through information posters around the surgery and batch SMS message updates.
- Specify requirements for improved training of reception staff in handling results. This would include guidance on communicating potentially sensitive information.
- Capitalise on the growth in use of patient messaging platforms such as Accurx[46], resulting from the COVID-19 pandemic, to develop formal pathways within General Practices for pro-actively communicating negative results and safety-netting advice:
    - Encourage direct SMS messaging of negative results to patients using pre-built templates which also contain information advising patients to contact their GP again if symptoms persist.
    - For those without a recorded mobile phone number, adopt a receptionist-led system of contacting the patient's landline, sending a letter informing them of their negative result if unable to get through.

Policy

- Instigate policy nationally to support standardisation of practices, such as those suggested above, within primary care.
- Ensure patients are included within the design and evaluation of future services and care models.

Conclusion

Overall, patients presenting in primary care with symptoms of possible CRC find FIT an acceptable test, even when facing challenges to collect their samples. Acceptability was influenced by personal knowledge and previous experience. FIT acceptability was high despite reports of poor experience of care, particularly regarding communication about the test and its results. There is scope for improving the test itself and its communication to enhance patient experience. Future studies should investigate patient acceptability and experience of care among those who choose not to do FIT, across different ethnic groups and different levels of social deprivation.

For peer review only

Figure legend

Figure 1. Themes and subthemes: patient experience and acceptability of using FIT

Acknowledgements

We thank all the patients who agreed to be interviewed. We thank the North East Essex and Suffolk Pathology Services at the East Suffolk and North Essex NHS Foundation Trust, and the Clinical Biochemistry section of Laboratory Medicine at Norfolk and Norwich University Hospitals NHS Foundation Trust. They have added a study recruitment letter, consent form and prepaid envelope to each FIT kit, distributed these to GP practices, and provided data on FIT results. We thank Prof Niek de Wit and Dr Peter Holloway for their support in the earlier stages of the study. We are grateful to Dr Marije van Melle for her work in the early stages of study design. We thank James Brimicombe for data management advice, support and expertise, and Andy Cowan for his help collecting and managing FIT-East study data. We are grateful to the CanTest Patient & Public Involvement (PPI) Panel (special thanks to Margaret Johnson) for reading and commenting on interview transcripts.

Supplemental material

Supplementary File 1. Interview topic schedule
Supplementary File 2. Interviews in context
COREQ checklist

Patient consent for publication

Not required.

Funding statement

This work was supported by the CanTest Collaborative, which is funded by Cancer Research UK grant number C8640/A23385, of which FMW and WH are co-Directors, and NC, MMP and SH are postdoctoral researchers.

This work was co-funded by the National Institute for Health Research (NIHR) Policy Research Programme, conducted through the Policy Research Unit in Cancer Awareness, Screening and Early Diagnosis, PR-PRU-1217-21601. The views expressed in this publication are those of the authors and not necessarily those of the National Health Service, the NIHR or the Department of Health. The funding sources had no role in the study design, data collection, data analysis, data interpretation, writing of the report or in the decision to submit for publication.

Competing interests

None declared.

For peer review only

Contributors

MMP and FMW planned the original study; WH and FMW secured funding. SH, SA and MMP carried out the interviews. Analysis was planned and carried out by CS, NC, SH, SA, AF, AC and FMW. CS and NC drafted the manuscript with senior input from SA and FMW and revised it after critical reviews from all authors. All authors read and approved the final manuscript.

Data sharing statement

Anonymised data are available upon reasonable request.

Ethics Approval Statement

Ethical approval was granted by the East of England Cambridge Central Research Ethics Committee – reference 19/EE/0036.

For peer review only

For peer review only - http://bmjopen.bmj.com/site/about/guidelines.xhtml

REFERENCES

1. Colorectal cancer statistics [Internet]. WCRF International. [cited 2023 Apr 14]. Available from: https://www.wcrf.org/cancer-trends/colorectal-cancer-statistics/

2. 1 Recommendations | Quantitative faecal immunochemical tests to guide referral for colorectal cancer in primary care | Guidance | NICE [Internet]. NICE; [cited 2022 Jan 21]. Available from: https://www.nice.org.uk/guidance/dg30/chapter/1-Recommendations

3. Young GP, Symonds EL, Allison JE, Cole SR, Fraser CG, Halloran SP, et al. Advances in Fecal Occult Blood Tests: The FIT Revolution. Dig Dis Sci. 2015;60(3):609–22.

4. Koo MM, von Wagner C, Abel GA, McPhail S, Hamilton W, Rubin GP, et al. The nature and frequency of abdominal symptoms in cancer patients and their associations with time to help-seeking: evidence from a national audit of cancer diagnosis. J Public Health Oxf Engl. 2018 Sep;40(3):e388–95.

5. Moreno CC, Mittal PK, Sullivan PS, Rutherford R, Staley CA, Cardona K, et al. Colorectal Cancer Initial Diagnosis: Screening Colonoscopy, Diagnostic Colonoscopy, or Emergent Surgery, and Tumor Stage and Size at Initial Presentation. Clin Colorectal Cancer. 2016 Mar;15(1):67–73.

6. Diagnosis | Gastrointestinal tract (lower) cancers - recognition and referral | CKS | NICE [Internet]. [cited 2022 May 19]. Available from: https://cks.nice.org.uk/topics/gastrointestinal-tract-lower-cancers-recognition-referral/diagnosis/

7. Overview | Suspected cancer: recognition and referral | Guidance | NICE [Internet]. NICE; [cited 2022 Jan 21]. Available from: https://www.nice.org.uk/guidance/ng12

8. Hamilton W. The CAPER studies: five case-control studies aimed at identifying and quantifying the risk of cancer in symptomatic primary care patients. Br J Cancer. 2009 Dec 3;101 Suppl 2:S80-86.

9. Bailey SER, Abel GA, Atkins A, Byford R, Davies SJ, Mays J, et al. Diagnostic performance of a faecal immunochemical test for patients with low-risk symptoms of colorectal cancer in primary care: an evaluation in the South West of England. Br J Cancer. 2021 Mar;124(7):1231–6.

10. Pin-Vieito N, Tejido-Sandoval C, Vicente-Bielza N de, Sánchez-Gómez C, Cubiella J. Faecal immunochemical tests safely enhance rational use of resources during the assessment of suspected symptomatic colorectal cancer in primary care: systematic review and meta-analysis. Gut. 2022 May 1;71(5):950–60.

11. The NHS Bowel screening programme | Background information | Bowel screening | CKS | NICE [Internet]. [cited 2022 Feb 1]. Available from: https://cks.nice.org.uk/topics/bowel-screening/background-information/the-nhs-bowel-screening-programme/#bowel-cancer-screening-programme-in-england

12. Joint ACPGBI, BSG and BSGAR considerations for adapting the rapid access colorectoral cancer pathway during COVID-19 pandemic [Internet]. The British Society of

For peer review only

Gastroenterology. 2020 [cited 2022 Jan 24]. Available from: https://www.bsg.org.uk/covid-19-advice/covid-19-advice-for-healthcare-professionals/joint-acgbbi-bsg-and-bsgar-considerations-for-adapting-the-rapid-access-colorectoral-cancer-pathway-during-covid-19-pandemic/

13. Maclean W, Limb C, Mackenzie P, Whyte MB, Benton SC, Rockall T, et al. Adoption of faecal immunochemical testing for 2-week-wait colorectal patients during the COVID-19 pandemic: an observational cohort study reporting a new service at a regional centre. Colorectal Dis. 2021;23(7):1622–9.

14. D'Souza N, Abulafi M. Navigating the storm of COVID-19 for patients with suspected bowel cancer. Br J Surg. 2020 May 13;107(7):e204.

15. Arasaradnam RP, Bhala N, Evans C, Greenaway J, Logan R, Penman I, et al. Faecal immunochemical testing in the COVID-19 era: balancing risk and costs. Lancet Gastroenterol Hepatol. 2020 Aug;5(8):717–9.

16. Faecal Immunochemical Testing (FIT) in patients with signs or symptoms of suspected colorectal cancer (CRC): A joint guideline from the Association of Coloproctology of Great Britain & Ireland (ACPGBI) and the British Society of Gastroenterology (BSG) [Internet]. The British Society of Gastroenterology. [cited 2022 Jun 10]. Available from: https://www.bsg.org.uk/clinical-resource/faecal-immunochemical-testing-fit-in-patients-with-signs-or-symptoms-of-suspected-colorectal-cancer-crc-a-joint-guideline-from-the-acpgbi-and-the-bsg/

17. Monahan KJ, Davies MM, Abulafi M, Banerjea A, Nicholson BD, Arasaradnam R, et al. Faecal immunochemical testing (FIT) in patients with signs or symptoms of suspected colorectal cancer (CRC): a joint guideline from the Association of Coloproctology of Great Britain and Ireland (ACPGBI) and the British Society of Gastroenterology (BSG). Gut [Internet]. 2022 Jul 12 [cited 2022 Aug 8]; Available from: http://gut.bmj.com/content/early/2022/07/25/gutjnl-2022-327985

18. Project information | Quantitative faecal immunochemical tests to guide colorectal cancer pathway referral for people with a change in bowel habit or abdominal pain | Guidance | NICE [Internet]. NICE; [cited 2022 Aug 8]. Available from: https://www.nice.org.uk/guidance/indevelopment/gid-dg10036

19. Chambers JA, Callander AS, Grangeret R, O'Carroll RE. Attitudes towards the Faecal Occult Blood Test (FOBT) versus the Faecal Immunochemical Test (FIT) for colorectal cancer screening: perceived ease of completion and disgust. BMC Cancer. 2016 Dec;16(1):1–7.

20. Bowyer HL, Vart G, Kralj-Hans I, Atkin W, Halloran SP, Seaman H, et al. Patient attitudes towards faecal immunochemical testing for haemoglobin as an alternative to colonoscopic surveillance of groups at increased risk of colorectal cancer. J Med Screen. 2013 Sep 1;20(3):149–56.

21. Chin YH, Ng CH, Seah SHY, Jain SR, Law JH, Devi MK, et al. Evolving perspectives on stool testing for colorectal cancer: a qualitative systematic review. Eur J Cancer Prev. 2020 Sep;29(5):416–23.

For peer review only

22. Johanna W, Yvonne W, Anna J, Kaisa F. Two sides of every coin: individuals' experiences of undergoing colorectal cancer screening by faecal immunochemical test and colonoscopy. Eur J Public Health. 2021 Dec 1;31(6):1290–5.

23. Vart G, Banzi R, Minozzi S. Comparing participation rates between immunochemical and guaiac faecal occult blood tests: a systematic review and meta-analysis. Prev Med. 2012 Aug;55(2):87–92.

24. Goodwin B, Viljoen B, Myers L, Ireland M, Dunn J, Chambers S, et al. Identifying modifiable features of home bowel cancer screening kits to facilitate use: consumer perspectives. Public Health Res Pract [Internet]. 2022 [cited 2022 Jun 27]; Available from: https://www.phrp.com.au/?p=41545

25. Delisle TG, D'Souza N, Davies B, Benton S, Chen M, Ward H, et al. Faecal immunochemical test for suspected colorectal cancer symptoms: patient survey of usability and acceptability. BJGP Open [Internet]. 2022 Jan 12 [cited 2022 Feb 8]; Available from: https://bjgpopen.org/content/early/2022/01/10/BJGPO.2021.0102

26. Forster AS, Rubin G, Emery JD, Thompson M, Sutton S, de Wit N, et al. Measuring patient experience of diagnostic care and acceptability of testing. Diagnosis. 2021 Aug 26;8(3):317–21.

27. Gil N, Su H, Kaur K, Barnett M, Murray A, Duffy S, et al. Patient experience and satisfaction with symptomatic Faecal Immunochemical Testing: An explanatory sequential mixed-methods evaluation. Br J Gen Pract [Internet]. 2022 Oct 18 [cited 2022 Oct 19]; Available from: https://bjgp.org/content/early/2022/10/18/BJGP.2022.0241

28. Calanzani N, Pannebakker M, Tagg MJ, Walford H, Holloway P, de Wit N,Hamilton W, Walter FM. Who are the patients being offered the faecal immunochemical test in routine English general practice, and for what symptoms? A prospective descriptive study. BMJ Open 2022. ((in press)).

29. Archer S, Calanzani N, Honey S, Johnson M, Neal R, Scott SE, et al. Impact of the COVID-19 pandemic on cancer assessment in primary care: a qualitative study of GP views. BJGP Open. 2021 Aug;5(4):BJGPO.2021.0056.

30. Tong A, Sainsbury P, Craig J. Consolidated criteria for reporting qualitative research (COREQ): a 32-item checklist for interviews and focus groups. Int J Qual Health Care. 2007 Dec 1;19(6):349–57.

31. Scott SE, Walter FM, Webster A, Sutton S, Emery J. The Model of Pathways to Treatment: Conceptualization and integration with existing theory. Br J Health Psychol. 2013;18(1):45–65.

32. Walter F, Webster A, Scott S, Emery J. The Andersen Model of Total Patient Delay: a systematic review of its application in cancer diagnosis. J Health Serv Res Policy. 2012 Apr;17(2):110–8.

33. Sekhon M, Cartwright M, Francis JJ. Acceptability of health care interventions: A theoretical framework and proposed research agenda. Br J Health Psychol. 2018;23(3):519–31.

For peer review only

34. Gale NK, Heath G, Cameron E, Rashid S, Redwood S. Using the framework method for the analysis of qualitative data in multi-disciplinary health research. BMC Med Res Methodol. 2013 Dec;13(1):117.

35. Best Qualitative Data Analysis Software for Researchers | NVivo [Internet]. [cited 2022 May 16]. Available from: https://www.qsrinternational.com/nvivo-qualitative-data-analysis-software/home

36. University of Cambridge | CanTest [Internet]. CanTest |. 2019 [cited 2022 Jun 7]. Available from: https://www.cantest.org/about/university-of-cambridge/

37. Regional ethnic diversity [Internet]. [cited 2022 Jun 23]. Available from: https://www.ethnicity-facts-figures.service.gov.uk/uk-population-by-ethnicity/national-and-regional-populations/regional-ethnic-diversity/latest

38. Vuik FE, Nieuwenburg SA, Bardou M, Lansdorp-Vogelaar I, Dinis-Ribeiro M, Bento MJ, et al. Increasing incidence of colorectal cancer in young adults in Europe over the last 25 years. Gut. 2019 Oct;68(10):1820–6.

39. Araghi M, Soerjomataram I, Bardot A, Ferlay J, Cabasag CJ, Morrison DS, et al. Changes in colorectal cancer incidence in seven high-income countries: a population-based study. Lancet Gastroenterol Hepatol. 2019 Jul 1;4(7):511–8.

40. Loomans-Kropp HA, Umar A. Increasing Incidence of Colorectal Cancer in Young Adults. J Cancer Epidemiol. 2019 Nov 11;2019:9841295.

41. Clarke N, Gallagher P, Kearney PM, McNamara D, Sharp L. Impact of gender on decisions to participate in faecal immunochemical test-based colorectal cancer screening: a qualitative study. Psychooncology. 2016;25(12):1456–62.

42. Litchfield IJ, Bentham LM, Lilford RJ, McManus RJ, Greenfield SM. Patient perspectives on test result communication in primary care: a qualitative study. Br J Gen Pract. 2015 Mar;65(632):e133–40.

43. Sampson R, Cooper J, Barbour R, Polson R, Wilson P. Patients' perspectives on the medical primary–secondary care interface: systematic review and synthesis of qualitative research. BMJ Open. 2015 Oct;5(10):e008708.

44. Banks J, Walter FM, Hall N, Mills K, Hamilton W, Turner KM. Decision making and referral from primary care for possible lung and colorectal cancer: a qualitative study of patients' experiences. Br J Gen Pract. 2014 Dec 1;64(629):e775–82.

45. Skivington K, Matthews L, Simpson SA, Craig P, Baird J, Blazeby JM, et al. A new framework for developing and evaluating complex interventions: update of Medical Research Council guidance. BMJ. 2021 Sep 30;374:n2061.

46. Accurx for Primary Care [Internet]. [cited 2022 Aug 8]. Available from: https://www.accurx.com/primary-care

For peer review only - http://bmjopen.bmj.com/site/about/guidelines.xhtml

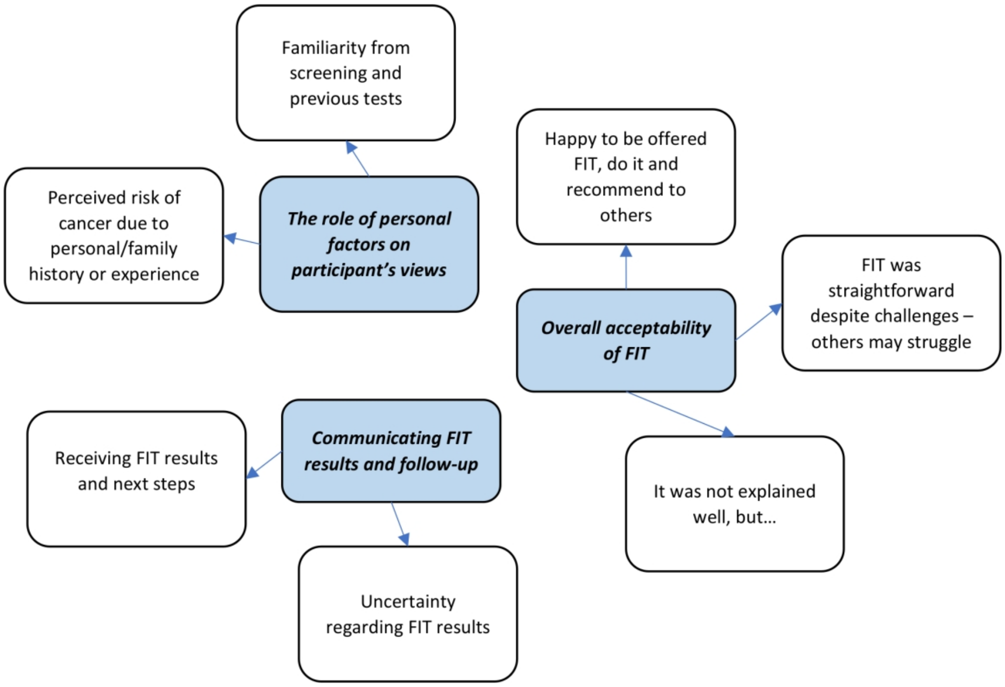

Figure 1. Themes and subthemes: patient experience and acceptability of using FIT

168x114mm (300 x 300 DPI)

Supplementary File 1. Interview topic schedule

Amended COVID items in italics

Background

- Tell me a bit about you:
    - age
    - education
    - employment history
    - daily activities
    - general health

- *What impact has coronavirus had on your everyday life?*

Background to FIT

- What was your overall experience of the problem, getting tested and receiving your test result?

- Tell me more about the symptoms you experienced
    - Abdominal discomfort
    - Change in bowel habit (general, diarrhoea, constipation)
    - Bleeding
    - Indigestion / heartburn
    - Bloating
    - Fatigue
    - Unexplained weight loss
    - Loss of appetite.

- How long had you experienced symptoms?
    - *Note: Try and get dates to be able to work out how this relates to COVID*

- Did you try any self-management?
    - *If yes, do you think coronavirus affected how you self-managed?*

- When did you first seek help for symptoms?
    - *Do you think coronavirus had any impact on when and where you sought help?*
        - *Friends and family*
        - *Other sources of information (e.g. internet)*
        - *Other medical professional (e.g. pharmacist)*
    - *Do you think coronavirus has changed the way you seek help for medical problems?*
        - *Changing thresholds for going to the doctors*
        - *Ongoing fear of going out*
        - *Change in the way that clinical appointments happen*

- Did you take any prescription medication for symptoms?
    - How long taking
    - Any changes to medication and/or dose
    - How medication has helped
    - Any problems?
    - *Do you think coronavirus led to any problems getting medication?*

Experience of GP consultation where the FIT-poo test was first suggested

- How they were feeling before GP appointment (about symptoms, urgency, what did they expect to happen)?

For peer review only

- *Did coronavirus have any impact on how you interacted with your GP?*
    - *Did you have a face to face appointment?*
    - *Were there any remote part to the consultation?*
    - *Was this what you were expecting?*

- *What was it like to going to the GP practice during coronavirus?*
    - *How did this make you feel?*
    - *How do you feel about attending any future appointments with your GP?*

- How did you feel when the GP suggested having a FIT-poo test?
    - How was the FIT-poo test introduced and explained?.
    - Who explained this (GP, nurse, admin?).
    - What did you think about the information you received (format and content).

- How did you feel following the GP consultation but before doing the test (physically and emotionally)?

- How long did you wait between the GP visit and doing the FIT-poo test at home?
    - What factors influenced this (busy/scared/anxious/collecting additional information?).
    - Did you discuss doing the FIT-poo test with anyone (family member, friend, GP, nurse)
    - What was your experience of discussion?
    - Did you look up or request any further information (including internet).
    - Any concerns about taking the test?

- Do you have any ideas about why people might decide against taking the test (why others might be put off)?

- What did you think the doctor was looking for?
    - Did you or they make any links between colorectal cancer and your these symptoms?

Experience of doing the FIT-poo test

- How did you feel about doing the test?
    - Physically
    - Emotionally
    - Did you manage first time you tried? If not, why not?

- How did you feel following the test?
    - Explore for immediately after test
    - Later that day
    - Following day
    - Over following week.
- *Do you think the coronavirus made you more/less concerned about the test?*

Experience of receiving the result of the FIT-poo test

- What was it like waiting for result?
    - *Do you think coronavirus affected this?*
    - For those who have not yet had a result – why do they think this might be?
    - How did you receive the test result (text message, letter, phone call, another consultation)
    - *Do you think coronavirus has had any impact on the timeliness/delivery of results?*

- What was it like being given a POSITIVE / NEGATIVE / EQIVOCAL result?

- Do you know what will happen next?
    - *Are there any concerns about future care because of coronavirus?*

For peer review only

- Do you think your experience has influenced any of your friends or family members about seeking/receiving care now and in the future?

Improvements to the FIT-poo test experience

- What went well before, during and after doing the FIT-poo test?
    - Any suggestions for changes that would improve FIT-poo test
        - Information provided
        - Procedure
        - Staff interpersonal skills
        - Experience of taking the FIT-poo test.

- *What impact, if any, did coronavirus have on your testing experience?*
    - *Has this changed their view of future care and what this might look like post-covid?*

- Would you take test again?

- Would you recommend test to someone else?
    - How would you explain test to someone else?

Ask whether anything else participant would like to mention / discuss

For peer review only

Supplementary File 2. Interviews in context: patients' experiences during the COVID-19 pandemic

Participants described feeling isolated from their support networks due to the limitations on social interactions during the pandemic. This resulted in some feeling unable to have conversations with family and friends regarding their symptoms during the appraisal period.

Many delayed seeking help as a result of what they perceived was happening within General Practice. A range of attitudes were displayed, from believing practices were overrun, to feeling GPs were 'shutdown' or only open for emergencies. For some, this resulted in the decision to seek help from an alternative provider such as 111 or walk-in services. In contrast, for a minority of participants positive media campaigns advising the public that the NHS remained open influenced them to contact their GP.

Changes to remote consulting (primarily in the form of telephone consultations) had both positive and negative impacts. Though many highlighted the convenience of telephone appointments and the speed of talking to their GP, others described the challenges of navigating complicated booking systems, and describing problems over the phone.

For those that visited their GP surgery during the pandemic, personal protective equipment and social distancing measures were generally expected and accepted, despite often feeling time-consuming or frustrating. Many perceived GP practices to be safer and cleaner than other public spaces.

For peer review only

COREQ (COnsolidated criteria for REporting Qualitative research) Checklist

A checklist of items that should be included in reports of qualitative research. You must report the page number in your manuscript where you consider each of the items listed in this checklist. If you have not included this information, either revise your manuscript accordingly before submitting or note N/A.

Topic	Item No.	Guide Questions/Description	Reported on Page No.
**Domain 1: Research team and reflexivity**			
Personal characteristics			
Interviewer/facilitator	1	Which author/s conducted the interview or focus group?	
Credentials	2	What were the researcher's credentials? E.g. PhD, MD	
Occupation	3	What was their occupation at the time of the study?	
Gender	4	Was the researcher male or female?	
Experience and training	5	What experience or training did the researcher have?	
Relationship with participants			
Relationship established	6	Was a relationship established prior to study commencement?	
Participant knowledge of the interviewer	7	What did the participants know about the researcher? e.g. personal goals, reasons for doing the research	
Interviewer characteristics	8	What characteristics were reported about the inter viewer/facilitator? e.g. Bias, assumptions, reasons and interests in the research topic	
**Domain 2: Study design**			
Theoretical framework			
Methodological orientation and Theory	9	What methodological orientation was stated to underpin the study? e.g. grounded theory, discourse analysis, ethnography, phenomenology, content analysis	
Participant selection			
Sampling	10	How were participants selected? e.g. purposive, convenience, consecutive, snowball	
Method of approach	11	How were participants approached? e.g. face-to-face, telephone, mail, email	
Sample size	12	How many participants were in the study?	
Non-participation	13	How many people refused to participate or dropped out? Reasons?	
Setting			
Setting of data collection	14	Where was the data collected? e.g. home, clinic, workplace	
Presence of non-participants	15	Was anyone else present besides the participants and researchers?	
Description of sample	16	What are the important characteristics of the sample? e.g. demographic data, date	
Data collection			
Interview guide	17	Were questions, prompts, guides provided by the authors? Was it pilot tested?	
Repeat interviews	18	Were repeat inter views carried out? If yes, how many?	
Audio/visual recording	19	Did the research use audio or visual recording to collect the data?	
Field notes	20	Were field notes made during and/or after the inter view or focus group?	
Duration	21	What was the duration of the inter views or focus group?	
Data saturation	22	Was data saturation discussed?	
Transcripts returned	23	Were transcripts returned to participants for comment and/or	

For peer review only

Topic	Item No.	Guide Questions/Description	Reported on Page No.
		correction?	
**Domain 3: analysis and findings**			
Data analysis			
Number of data coders	24	How many data coders coded the data?	
Description of the coding tree	25	Did authors provide a description of the coding tree?	
Derivation of themes	26	Were themes identified in advance or derived from the data?	
Software	27	What software, if applicable, was used to manage the data?	
Participant checking	28	Did participants provide feedback on the findings?	
Reporting			
Quotations presented	29	Were participant quotations presented to illustrate the themes/findings? Was each quotation identified? e.g. participant number	
Data and findings consistent	30	Was there consistency between the data presented and the findings?	
Clarity of major themes	31	Were major themes clearly presented in the findings?	
Clarity of minor themes	32	Is there a description of diverse cases or discussion of minor themes?	

Developed from: Tong A, Sainsbury P, Craig J. Consolidated criteria for reporting qualitative research (COREQ): a 32-item checklist for interviews and focus groups. *International Journal for Quality in Health Care*. 2007. Volume 19, Number 6: pp. 349 – 357

Once you have completed this checklist, please save a copy and upload it as part of your submission. DO NOT include this checklist as part of the main manuscript document. It must be uploaded as a separate file.

For peer review only

Can we do better? A qualitative study in the East of England investigating patient experience and acceptability of using the faecal immunochemical test in primary care

Journal:	*BMJ Open*
Manuscript ID	bmjopen-2023-072359.R2
Article Type:	Original research
Date Submitted by the Author:	26-May-2023
Complete List of Authors:	Snudden, Claudia; University of Cambridge Department of Public Health and Primary Care,<br>Calanzani, Natalia; University of Aberdeen Institute of Applied Health Sciences, Academic Primary Care; University of Cambridge Department of Public Health and Primary Care<br>Archer, Stephanie; University of Cambridge Department of Public Health and Primary Care<br>Honey, Stephanie; University of Leeds Leeds Institute of Health Sciences<br>Pannebakker, Merel; University of Cambridge Department of Public Health and Primary Care<br>Faher, Anissa; University of Cambridge School of Clinical Medicine<br>Chang, Aina; University of Cambridge School of Clinical Medicine<br>Hamilton, Willie; University of Exeter<br>Walter, Fiona M; Queen Mary University of London, Wolfson Institute of Population Health; University of Cambridge, Department of Public Health and Primary Care
**Primary Subject Heading**:	General practice / Family practice
Secondary Subject Heading:	Oncology
Keywords:	PRIMARY CARE, QUALITATIVE RESEARCH, GASTROENTEROLOGY

SCHOLARONE™
Manuscripts

I, the Submitting Author has the right to grant and does grant on behalf of all authors of the Work (as defined in the below author licence), an exclusive licence and/or a non-exclusive licence for contributions from authors who are: i) UK Crown employees; ii) where BMJ has agreed a CC-BY licence shall apply, and/or iii) in accordance with the terms applicable for US Federal Government officers or employees acting as part of their official duties; on a worldwide, perpetual, irrevocable, royalty-free basis to BMJ Publishing Group Ltd ("BMJ") its licensees and where the relevant Journal is co-owned by BMJ to the co-owners of the Journal, to publish the Work in this journal and any other BMJ products and to exploit all rights, as set out in our licence.

The Submitting Author accepts and understands that any supply made under these terms is made by BMJ to the Submitting Author unless you are acting as an employee on behalf of your employer or a postgraduate student of an affiliated institution which is paying any applicable article publishing charge ("APC") for Open Access articles. Where the Submitting Author wishes to make the Work available on an Open Access basis (and intends to pay the relevant APC), the terms of reuse of such Open Access shall be governed by a Creative Commons licence – details of these licences and which Creative Commons licence will apply to this Work are set out in our licence referred to above.

Other than as permitted in any relevant BMJ Author's Self Archiving Policies, I confirm this Work has not been accepted for publication elsewhere, is not being considered for publication elsewhere and does not duplicate material already published. I confirm all authors consent to publication of this Work and authorise the granting of this licence.

For peer review only

Can we do better? A qualitative study in the East of England investigating patient experience and acceptability of using the faecal immunochemical test in primary care

Claudia M Snudden[1] BMBCh (ORCID ID: 0000-0002-7355-4317)
Natalia Calanzani[1,2] PhD (ORCID ID: 0000-0002-5068-2543)
Stephanie Archer[1] PhD (ORCID ID: 0000-0003-1349-7178)
Stephanie Honey[3] PhD (ORCID ID: 0000-0002-2675-7824)
Merel M Pannebakker[1] PhD (ORCID ID: 0000-0003-2918-0570)
Anissa Faher[4] BA Hons (Cantab)
Aina Chang[4] BA Hons (Cantab)
Willie Hamilton[5] FRCGP (ORCID ID: 0000-0003-1611-1373)
Fiona M Walter[6,1] FRCGP (ORCID ID: 0000-0002-7191-6476)

[1] The Primary Care Unit, Department of Public Health and Primary Care, University of Cambridge, Cambridge, UK
[2] Academic Primary Care, Institute of Applied Health Sciences, University of Aberdeen, Aberdeen, UK
[3] Leeds Institute of Health Sciences, University of Leeds, Leeds, UK
[4] School of Clinical Medicine, Addenbrooke's Hospital, University of Cambridge, UK
[5] University of Exeter Medical School, Exeter, UK
[6] Wolfson Institute of Population Health, Queen Mary University of London, UK

Corresponding author: Dr Claudia Snudden
Email: cs2048@medschl.cam.ac.uk

Address: The Primary Care Unit, Department of Public Health and Primary Care, University of Cambridge, Cambridge, UK

Word count: 4438

For peer review only

ABSTRACT

Objectives

The faecal immunochemical test (FIT) is increasingly used in UK primary care to triage patients presenting with symptoms and at different levels of colorectal cancer risk. Evidence is scarce on patients' views of using FIT in this context. We aimed to explore patients' care experience and acceptability of using FIT in primary care.

Design

A qualitative semi-structured interview study. Interviews were conducted via Zoom between April and October 2020. Transcribed recordings were analysed using framework analysis.

Setting

East of England General Practices.

Participants

Consenting patients (aged ≥40 years) who presented in primary care with possible symptoms of colorectal cancer, and for whom a FIT was requested, were recruited to the FIT-East study. Participants were purposively sampled for this qualitative sub-study based on age, gender, and FIT result.

Results

44 participants were interviewed with a mean age 61 years, and 25 (57%) being male: 8 (18%) received a positive FIT result. Three themes and seven subthemes were identified. Participants' familiarity with similar tests and perceived risk of cancer influenced test experience and acceptability. All participants were happy to do the FIT themselves and to recommend it to others. Most participants reported that the test was straightforward, although some considered it may be a challenge to others. However, test explanation by healthcare professionals was often limited. Furthermore, while some participants received their results quickly, many did not receive them at all with the common assumption that "no news is good news". For those with a negative result and persisting symptoms, there was uncertainty about any next steps.

For peer review only

Conclusions

While FIT is acceptable to patients, elements of communication with patients by the healthcare system show potential for improvement. We suggest possible ways to improve the FIT experience, particularly regarding communication about the test and its results.

For peer review only

For peer review only - http://bmjopen.bmj.com/site/about/guidelines.xhtml

STRENGTHS AND LIMITATIONS OF THIS STUDY

- Interested participants were contacted within four weeks of performing FIT, helping to reduce recall bias
- All participants were White (British or other) and English speakers – views may differ for those identifying with other ethnicities or who do not understand English well
- The use of Zoom for participant interviews may have hindered those unable to navigate the required technology from taking part

For peer review only

INTRODUCTION

Colorectal cancer (CRC) is the third most common cancer worldwide and incidence is increasing[1]. Globally, mortality rates are decreasing, in part due to increased screening, improved early diagnosis, and advances in treatment[2,3]. Within the United Kingdom (UK), the majority of patients diagnosed with CRC will first present to their General Practitioner (GP) with abdominal symptoms[4,5]. GPs act as gatekeepers within the UK's National Health Service (NHS), deciding which patients require onwards referrals to secondary care, and how urgently.

Diagnosing CRC, particularly in its early stages, can be difficult as symptoms of CRC are broad and non-specific[6]. GPs in England currently follow National Institute for Health and Care Excellence (NICE) 2015 guidelines for patients with suspected cancer to identify higher risk patients who should be urgently referred for further investigation[7]. In 2017, NICE recommended the use of the Faecal Immunochemical Test (FIT) within primary care to help triage low risk patients with possible CRC symptoms that do not meet urgent referral criteria[2,8]. FIT can be used for both ruling in and ruling out CRC[9,10]. Alongside its use in symptomatic patients, FIT is also used in the UK's bowel cancer screening programme, having replaced the previously used guaiac faecal occult blood test (gFOBT)[11].

The threshold set to define a positive FIT result (i.e., the haemoglobin cut-off concentration) differs between populations. For symptomatic patients, NICE advises that those with a FIT result ≥ 10μg Hb/g faeces are referred on for further diagnostic testing; of these about 7% will be diagnosed with CRC[2,9]. In contrast, England uses a cut-off of 120μg Hb/g in asymptomatic screening to select who requires further investigation[11]. Variations in thresholds are due to the differences in risk between asymptomatic and symptomatic populations; the risk of cancer is higher for those with symptoms therefore the threshold for further investigations is lower.

As a result of the COVID-19 pandemic, FIT use in primary care was intensified to help prioritise urgent referrals[12–15]. In June 2022, The British Society of Gastroenterology released additional guidelines recommending the continued use of FIT as a diagnostic triage tool for all urgent referrals[16,17]. NICE also plans to publish additional guidance on FIT by November 2023[18]. Given the increasing reliance of FIT use in primary care, it is critical to understand what patients think of the test and whether they find it acceptable. Acceptability is defined as a multi-faceted construct comprising affective attitude, burden, perceived effectiveness, ethicality, intervention coherence, opportunity costs, and self-efficacy[19]. It is a necessary condition for the

For peer review only - http://bmjopen.bmj.com/site/about/guidelines.xhtml

effectiveness of an intervention or test; from the patient perspective, if a test is considered acceptable, patients are more likely to adhere to the proposed investigation which in turn results in improved clinical outcomes[19,20].

Studies conducted in screening contexts have shown that disgust associated with performing stool tests and certain procedural aspects of FIT can present barriers to uptake, however, the test is often found acceptable[21–26]. Nonetheless, there is still limited research into FIT experience and acceptability, particularly within the context of symptomatic patients[27]. Understanding both care experience and acceptability is vital to ensure good quality of care for patients[28].

Since symptomatic populations have varied (and higher) levels of risk compared to an asymptomatic screening population, evidence on the patients' experience of care when and after doing FIT is crucial. To our knowledge, only two other studies have reported on symptomatic patients' views of FIT; one quantitative study focusing on usability and acceptability[27], and one mixed-methods study evaluating patient experience and satisfaction[29].

Therefore, this study aims to build on existing literature, providing an in-depth qualitative exploration of both FIT experience and acceptability for patients presenting to primary care with possible symptoms of CRC.

For peer review only

METHODS

Design, setting and population

This qualitative sub-study was undertaken as part of the FIT-East study, which was set across General Practices in the East of England Cancer Alliance (Suffolk & North East Essex and Norfolk & Waveney)[30]. FIT-East included 507 patients aged 40 years and older with possible symptoms of CRC, but who did not immediately meet urgent referral criteria, for whom a FIT was requested by the GP[30]. FIT kits contained a study recruitment letter and consent form; participants could tick a box if interested in taking part in interviews.

We undertook semi-structured interviews with patients to investigate experience and acceptability of using FIT in primary care. Interviews were also carried out with General Practitioners (reported elsewhere[31]). We followed the Consolidated Criteria for Reporting Qualitative studies (COREQ) guidelines[32]. Ethical approval was granted by the East of England Cambridge Central Research Ethics Committee – reference 19/EE/0036.

Sampling

We undertook purposive sampling based on age, gender, and FIT result to obtain a range of experiences among patients who consented to take part in the FIT-East study. Potential participants were contacted by email (provided by patients in the consent forms). To reduce recall bias, we contacted patients within four weeks of FIT being sent to the laboratory.

Ninety-nine participants responded positively to the initial email and were invited for interview. Additional informed consent was required and obtained for all patients. Interviews were carried out until no new topics were discussed: 45 participants were interviewed, mean duration 39.5 minutes. Due to the COVID-19 pandemic, interviews were carried out remotely using the Zoom platform, and audio-recorded. One interview could not be analysed due to a technical fault during recording; therefore 44 participants were included. Each participant was individually interviewed once by one or two experienced female health services researchers with a background in Psychology (SA and MMP) or Nursing (SH).

Interview topic guide

The guide (Supplementary File 1) explored patient experiences of being asked to undertake a FIT in primary care, covering areas including: obtaining the test, using it, returning it, and

For peer review only - http://bmjopen.bmj.com/site/about/guidelines.xhtml

receiving the results. The topic guide was piloted by the research team before use. In order to better understand patients' experiences during the COVID-19 pandemic, additional questions were added in June 2020. Interviews were carried out between April and October 2020.

Data analysis

Verbatim transcripts were checked, anonymised and analysed using framework analysis[33]. This allows for both inductive and deductive approaches. An inductive approach was adopted for data analysis, influenced by our research question and specific definitions of acceptability[33].

Four researchers (one academic GP [CS], one health services researcher [NC], and two medical students [AF and AC]) repeatedly read the transcripts and coded the interviews. Eight interviews were initially coded inductively by CS to discover potentially unexpected aspects of the participants' experiences. An analytical framework was then developed using codes from the eight initial transcripts; codes were grouped into categories, influenced by the relevant aspects of three theoretical models (Box 1). The framework was then used to systematically index the remaining transcripts, including double coding of a quarter of the data set. Codes and categories were constantly refined during analysis. Data were charted into a series of matrices from which themes were developed. Consistency of coding was discussed regularly during team meetings; themes were also amended with guidance from senior team members (SA and FMW). NVivo 12 was used to facilitate analysis[34].

Participant age (range), gender (M for male and F for female) and FIT results (positive, negative or unclear) are reported alongside quotes to aid interpretation of findings. Quotes were chosen to illustrate findings while also ensuring a good spread of participants and their characteristics.

Box 1. Theoretical models

Three theoretical models, listed below, were used to help inform and conceptualize the themes during data analysis. These models were chosen to underpin important complementary but distinctive aspects of the cancer diagnostic pathway[35,36] including the role of patient factors/characteristics[35,36], the construct of acceptability[19] and patient's experience of care[28].

1. The Model of Pathways to Treatment[35,36] describes events, processes, intervals and contributing factors (such as patient experience) underpinning the pathway to diagnosing cancer for patients presenting with symptoms.
2. The Theoretical Framework of Acceptability (TFA)[19] describes seven constructs of acceptability: affective attitude, burden, perceived effectiveness, ethicality, intervention

For peer review only

coherence, opportunity costs, and self-efficacy.
3. Forster's *et al* considerations when assessing acceptability of diagnostic tests[28] describes dimensions such as patient-centred care, continuity and coordination of care, and waiting times.

Patient and public involvement

Patient-public partners from the CanTest Collaborative[37] (responsible for FIT-East) provided feedback on the original and amended interview topic guides. Representatives also read and commented on interview transcripts; these comments were considered when iteratively revising our analysis.

For peer review only

For peer review only - http://bmjopen.bmj.com/site/about/guidelines.xhtml

RESULTS

Table 1 describes characteristics of the included participants and describes how FIT results were received. About a fifth (18%) had a positive FIT result. Outcome data for whether participants received a cancer diagnosis was not available.

Table 1. Demographic characteristics and FIT results (n=44)

Characteristic	Variable	N	%
Sex	Female	19	43
	Male	25	57
Age (years)	40-49	3	7
	50-59	17	39
	60-69	10	23
	70-79	11	25
	80-89	3	7
FIT result	Negative	35	80
	Positive	8	18
	Unclear*	1	2
How FIT result was received	Patient called practice†	12	27
	Patient did not receive results/is unsure‡	11	25
	Practice called patient§	9	20
	Patient told face-to-face‖	4	9
	Patient received letter¶	3	7
	Patient checked results online¶	2	5
	Other**	3	7
Education level	None	3	7
	GCSE (or equivalent)	4	9
	A level (or equivalent)	5	11
	Degree (or equivalent)	18	41
	Other higher education	10	23
	Missing	4	9
Ethnicity	White British	35	80
	White Irish	1	2
	Any other White background	2	4
	Missing	6	14
Indices of Multiple Deprivation (IMD) quintiles	1 (most deprived)	0	0
	2	4	9
	3	7	16
	4	4	9
	5 (least deprived)	6	14
	Missing	23	52

	* One patient described doing the FIT in detail, but on the patient database FIT was recorded as "not done". As the patient clearly had done the test, this was recorded as an unclear result.<br>†One positive result (patient reported this as inconclusive during the interview).<br>‡Did not receive (n=7, all negative), unsure about it (n=4, 2 negative, one positive and one missing)<br>§GP (n=6, 2 positive results), nurse practitioner (n=1, negative – nurse called about something else and the patient asked for results); receptionist (n=2, 1 positive result).<br>‖GP (n=2, all negative), nurse (n=1, positive), receptionist (n=1, negative)<br>¶ All negative results<br>**Cannot remember how results were received (n=1, positive), GP contacted but unclear if by phone or face-to-face (n=1, negative), over the phone but unclear who called whom (n=1, positive).

Overview and Context

We identified three themes and seven subthemes describing patient experience and acceptability of using FIT after presenting in primary care with possible symptoms of CRC (but not meeting immediately criteria for urgent referral) (Figure 1).

As the study was performed during the COVID-19 pandemic a brief description of COVID-19 related themes is available for context (Supplementary file 2). The pandemic did not appear to influence participants' experiences of using FIT, but it did affect how participants accessed their GP prior to receiving the test.

The role of personal factors on participant's views

Perceived risk of cancer due to personal/family history or experience

A personal history of having had cancer in the past encouraged participants to seek help quickly for their symptoms. Similarly, cancer experiences from family and friends influenced their decision to seek help after noticing body changes, and to do the test when it was offered:

> *"My father has had bowel cancer. He basically had it once and they took a section of the colon out. After a few years it returned so they removed the whole of his colon and he's now been…you know, God bless he's still alive and it's been sort of over 15 years he's been clear but there is that sort of history that I was aware of. So I thought, alright, better…you know, because it had changed slightly, yes, and it was bleeding more often, I thought I'd better go and get it looked at."* (PT18, M, 50-59y, negative)

Participants became concerned that their symptoms could be a sign of bowel cancer - either from their own knowledge of 'red flag' symptoms or from reading about symptoms online, leading them to actively seek advice. Others became increasingly anxious or concerned about

For peer review only - http://bmjopen.bmj.com/site/about/guidelines.xhtml

the severity of their symptoms, particularly if things had worsened over time or were causing significant discomfort.

On the other hand, having had a relatively recent examination or negative investigation (such as colonoscopy) often reassured them there was nothing serious and being offered a FIT was not considered to be worrying or concerning.

Familiarity from screening and previous tests

Participants were familiar with doing stool tests, usually as part of bowel screening or previous diagnostic processes. As a result, they often displayed no concerns with the idea of doing FIT:

> *"Well, I've been doing them for years. It didn't upset me, if that's what you mean. It's just one more thing, going to the bog isn't it? Going to the loo? Except this time you put it on a piece of […] paper, and get a smear and stick it in the packet, and then seal it up."* (PT1, M, 70-79y, negative)

Participants who had had previous experiences within secondary care (such as colonoscopies due to diverticular disease) described the FIT experience as less daunting:

> *"I was quite relaxed about* [being asked to do FIT] *because I'd had a colonoscopy before, that was five years ago, so that was more nerve-racking."* (PT25, F, 60-69y, negative)

Overall acceptability of FIT

Happy to be offered FIT, do it and recommend it to others

Overall, participants reported they were glad or relieved to be offered FIT and accepted the need to do it. This was true even when the potential unpleasantness or difficulties of doing a FIT were recognised. For some, doing a test could lead to an 'earlier diagnosis' and the potential to start treatment sooner if there was something serious going on. For others, acceptance was associated with wanting to find an answer to their symptoms, especially if these had been particularly distressing:

For peer review only - http://bmjopen.bmj.com/site/about/guidelines.xhtml

> *"I guess I felt a little bit strange, kind of, like here I am, you know, lining the toilet to catch my stools. But when you know that it's something that you want to get done and want to get answers for you just get on and do it really."* (PT12, F, 40-49y, negative)

When reflecting on why *others* might not want to do FIT, common reasons included the fear of a potential cancer diagnosis and finding the process disgusting or embarrassing:

> *"Probably because it's not a very nice thing to have to do, you know. Playing around with poo is not something that everybody thinks, 'oh that sounds like fun'."* (PT3, F, 60-69y, negative)

Every participant reported being happy to repeat FIT if necessary, and all would willingly recommend FIT to others if they needed it.

FIT was straightforward despite challenges – others may struggle

Participants commented on challenges they faced when physically collecting the FIT sample: often these were difficulties writing the label, 'catching' the stool sample without it falling into the toilet water or when suffering with diarrhoea, and finding the FIT stick small or *"very fiddly"* to handle.

Anxiety was also expressed regarding taking the FIT sample and "*wanting to do it right*". Furthermore, participants reported wanting to get the sample done and feeling relieved after completing the test:

> *"I wanted to get it out of the way, it's not exactly difficult, but it was just the having to, you know, deal with it. So I woke up thinking, 'oh, here we go, you know, I need to do this' and when I'd got things in the specimen jars and I sorted myself out, I was glad it was over."* (PT23, F, 50-59y, negative)

Methods to overcome difficulties collecting the sample were also described, drawing on prior experiences when completing bowel cancer screening tests. Nonetheless, participants (even those reporting these challenges) were confident about their ability to do the test, often remarking how simple and 'straightforward' FIT was.

For peer review only - http://bmjopen.bmj.com/site/about/guidelines.xhtml

In particular, participants found the instructions generally easy to follow. Nonetheless, they discussed why *others* might struggle with FIT – from potential difficulties faced by having arthritic or shaky hands, poor eyesight, or an upset stomach, to issues following instructions.

It was not explained well, but…

Participants agreed that the explanations given by their healthcare professional (usually a GP or practice nurse) regarding what FIT was and why it was indicated, tended to be poor:

> *"My GP didn't, actually, tell me what it was. I had to Google it to even find out what it was. He said 'we'll leave you out a FIT test', and I had no idea what it was."* (PT14, M, 60-69y, positive)

Nonetheless, some defended the professionals in such situations, attributing the limited information to common constraints in a consultation such as limited time to go through different medical problems.

Often participants were asked to do multiple stool tests as part of their symptom work-up, leading to confusion regarding the differences between tests. Frequent references were made to being handed a bag containing a FIT and being told to just follow the instructions. For two participants, the poor communication even resulted in confusion of the test with the similarly named Fitbit watch.

Whilst the text explanation was often inadequate, participants had very good intuition about linking FIT to CRC. Participants reported that GPs alluded to cancer but did not use the word 'cancer' specifically, choosing instead to describe it as "*something sinister*" or "*something nasty lurking*". Despite this, many appeared to interpret the GP's language appropriately:

> *"When he suggested doing a FIT test, which he explained to me what it was, I thought 'yeah, it makes sense really because I know what you're after'. He didn't tell me what he was after, but I thought 'I know what you're looking for'."* (PT24, M, 50-59y, negative)

Participants' understanding of how FIT worked varied considerably. The most common explanations focused around looking for traces of (often non-visible) blood in the stool, but others described FIT as "*a bit of an MOT check*", being used to "*determine the health of the*

For peer review only - http://bmjopen.bmj.com/site/about/guidelines.xhtml

gut", "*check the digestive system is working properly*" or look for "*abnormalities within the poo*". The internet was sometimes used to further research FIT and causes of blood in the stool.

Whilst most participants demonstrated good intuition or a basic understanding of FIT, some had no awareness as to why they were asked to do FIT, yet did it anyway:

> *"I just got given the test to do and, I suppose, in a roundabout, sort of, way, she was saying, you know, 'you need to have the test done', but I didn't understand it and I was puzzled about it [...] I think that's probably why I kept reading the notes about it, because I kept thinking, 'well, I'm not sure why I'm doing this'."* (PT33, F, 50-59y, negative)

Participants also talked about trusting the GP and their advice, and how this influenced their decision to do the FIT, how it would affect their decision to do it again or recommend it to others:

> *"I like her, I think she's an excellent GP. If she asks me to do something, to participate or provide a test for example, I trust her to be asking that for a good reason and I will instinctively comply with any requests for tests or anything else as I'm assuming that it's going to provide information, test results or whatever which will be for my direct benefit."* (PT19, M, 60-69y, negative)

Communicating FIT results and follow-up

Uncertainty regarding FIT results

Participants reported contrasting attitudes while waiting for test results. Some discussed the anxiety surrounding the waiting period, at times hypothesising what would happen next in the worst-case scenario.

In contrast, others were unaffected by waiting, seemingly managing to *"forget about it"* and carry on with life as usual. This was often linked to either normalising FIT to a more 'standard' test "*just like having a blood test*", or expecting to receive a negative result and therefore not seeing reason to worry.

Participants felt they weren't given clear information by the GP on when and how to access their results. They suggested that chasing results should be practice-led rather than patient-led,

For peer review only - http://bmjopen.bmj.com/site/about/guidelines.xhtml

regardless of the result. Some participants did not receive their results at all, others contacted the practice for these, and others assumed that the practice would only contact them if there was a positive result and reported not being worried as *"no news is good news"*:

> *"I haven't actually been told anything so I presume they were negative or there were no concerns because no GP has contacted me."* (PT20, F, 50-59y, negative)

Receiving FIT results and next steps

For those who received their test results, there were often comments on receiving these quickly. Nonetheless, the way results were communicated was not always well received, both for positive and negative results:

> *"The receptionist at the GP surgery telephoned me and said the doctor had referred me[...] and I said, 'oh, why is that then', and she...she didn't seem to know and I said 'I just had a FIT test, what was the result of that'? Pause, and she said, 'oh, yes, it was positive. I expect that's why it is'. And I'm not joking, that's exactly how the conversation went.*" (PT14, M, 60-69y, positive)

> *"I think one slightly disappointing aspect I suppose is that when I phoned for results, I only spoke to the receptionist, and all they were able to tell me was no further action necessary. And I kind of thought, 'oh well, it's great in one way and I was pleased with that, but it kind of didn't explain why I'd had problems earlier' and I thought, 'oh, do I just wait to see if I ever have this problem again'."* (PT41, M, 50-59y, negative)

For participants with a positive result, there were reported delays in having a colonoscopy, particularly during the COVID-19 pandemic. Participants were often stoical about this situation. For those with a negative result, a sense of relief and reassurance was frequently reported. However, when symptoms persisted, there was uncertainty regarding what to do next. Some said they would have valued a follow up consultation with their GP, but in practice this rarely happened, leaving participants feeling their problems had not been fully resolved. Others believed that the GP would not be able to offer them anything further even if they did arrange a further appointment:

For peer review only - http://bmjopen.bmj.com/site/about/guidelines.xhtml

"No, I don't know if there's a follow-up or anything, if you detect nothing does it just get swept under the carpet or do I stay on your database and do you check up again or…I don't know?" (PT6, M, 60-69y, negative)

For peer review only

DISCUSSION

Summary

This study of patients presenting in primary care with symptoms of possible CRC but not immediately meeting criteria for urgent referral found FIT an acceptable and straightforward test, despite some challenges manipulating the test kit. Participant characteristics such as past test experiences and perceived personal risk of cancer influenced attitudes towards FIT. Participants were also happy to do FIT again and recommend it to others, and trusted that their GP knew what was best for them. On the other hand, test explanations from health professionals were generally poor. This could be due, in part, to the lack of clarity surrounding FIT implementation and diagnostic pathways within primary care during the COVID-19 pandemic. There was also reported dissatisfaction with the way results were received, for both positive and negative results. Many participants did not receive results at all, and several assumed that "*no news is good news*". For some, there was no follow-up after a negative result, even when symptoms persisted.

Strengths and limitations

To our knowledge, this is the first qualitative study to explore experience and acceptability of FIT for symptomatic patients. We provide rich data on patients' care experiences and acceptability of FIT use in primary care. With the increased use of FIT in this setting, it is essential to understand patient views on the test and identify areas where care needs to be improved. Three well-established theoretical models were used to guide conceptualisation of the themes during analysis (Box 1)[19,28,35,36]. We found that the models complemented each other, with overlap between different elements. Patient factors[35] such as perception of risk, previous knowledge and experience influenced test acceptability[19], including the perceived burden, understanding why the test was being done (intervention coherence) and perceived ability to do the test (self-efficacy). Conversely, the overall test acceptability contrasted with the often reported less than ideal experience of care[28] before, during and after doing the test. Using a single model alone would have resulted in missing several nuances regarding acceptability, the role of patient factors and the quality of the experienced care.

Interested participants were contacted within four weeks of performing FIT, helping to reduce recall bias. There was good variation amongst participants in terms of age, gender and FIT result. However, only a sample of patients who took part in the FIT-East study were interviewed;

For peer review only - http://bmjopen.bmj.com/site/about/guidelines.xhtml

views of those who did not participate in the interviews may differ. The sample was also limited to the East of England. All participants identified themselves as White, slightly higher than the proportion reported by the UK Census for this region[38], and understood English well. Experiences of patients identifying with other ethnicities, and of those who do not speak English well may differ and need to be further investigated. Limited evidence on FIT use in symptomatic patients indicates that ethnicity may influence patient willingness to do the test[27]. Finally, we were not able to explore variations in views based on different levels of socioeconomic deprivation due to large amounts of missing data (52%). The relationship between poor care experience and test acceptability may differ for these groups; different levels of knowledge and experience may also play a role on FIT acceptability.

Due to resulting restrictions from the pandemic, interviews were conducted over Zoom, hindering those unable to navigate the required technology from taking part., No patients aged under 40 were recruited (as defined by our inclusion criteria). As FIT becomes more widely used in primary care, it will be key to explore the attitudes of this group, less familiar with stool testing and without involvement in prior screening programmes – particularly as the incidence of CRC is increasing in the under 50s[39–41].

Comparison with existing literature

Consistent with existing literature (although mostly investigating asymptomatic populations), FIT is seen as relatively straightforward with clear instructions[23–25,27]. Nonetheless, similar challenges to collecting the sample were reported including difficulties writing the label and handling the FIT stick[24,26]. Familiarity with stool tests is associated with higher intentions to use related tests in future, aligning with the confidence displayed by participants with experience of screening[27]. Social influences have also been associated with encouragement of FIT uptake, impacting an individual's perceived risk of, or susceptibility to, cancer[23,42].

Participants displayed different levels of proactivity in seeking FIT results, reflective of behaviours seen in the literature[43]. Patients reported wanting further clarification on how results are communicated; existing uncertainties were anxiety-inducing at times[24,43,44]. To our knowledge, there is no other available evidence on follow-up (or lack thereof) of patients with persisting symptoms and a negative FIT result.

Implications for research, practice and policy

For peer review only - http://bmjopen.bmj.com/site/about/guidelines.xhtml

Building on our findings and published literature, alongside considerations on what is feasible within the UK context, we have developed recommendations for research, practice and policy to improve both the FIT experience and patient safety (Box 2). Rationale for these recommendations is described below.

While FIT acceptance was high, there are still opportunities for improvement, particularly regarding processes to collect the stool sample, and better understanding FIT acceptability across different population groups.

Reports of questionable care about explaining the test, providing results and following up are also concerning[45]. While participants still found FIT acceptable and trusted that the GP knew what was good for them, care experiences caused anxiety to patients and indicate risks regarding continuity of care. Time waiting for results did not seem to be a problem for those who got them; but many reported that they never received the results. While no news was indeed often good news, some patients who chased their results found out they were positive. It is vital that GPs are clearer about when results are expected and how to access them, and safety-netting measures are in place to avoid missing cancers and provide better support to patients.

Finally, our results can support policy makers regarding FIT use and implementation within primary care. While flexibility and adaptability are required in the provision of services so different needs can be met[46], it is vital that national policies define standards on acceptable levels of service, particularly regarding how and when information on test results are provided.

Box 2. Recommendations for practice, research and policy

Research

- Investigate 1) challenges in collecting the stool sample, particularly for older people and those with mobility issues; and 2) improvements to sample collection (such as providing a collection container) and instructions (such as clarification on what to do in the case of diarrhoea).
- Investigate whether patients' presenting symptoms (high vs low risk) influence their perception of FIT, as we found that perceived risk and susceptibility influenced acceptability
- Seek to understand the views of patients who choose not to complete FIT after being offered it, to determine 1) why FIT may not be acceptable to some and 2) whether reasons align with issues identified in this study (e.g., test burden and limited information).
- Investigate the views of those living in areas of higher deprivation, and those identifying with different ethnic groups other than White.

Practice

For peer review only - http://bmjopen.bmj.com/site/about/guidelines.xhtml

- Work to improve GP communication with patients regarding why FIT is being done, what it looks for, and what a positive result means. Clear communication can also concurrently reduce patient confusion when multiple tests are requested simultaneously.
- Consider implementing a specific time slot for calling for results and promote these changes to patients through information posters around the surgery and batch SMS message updates.
- Specify requirements for improved training of reception staff in handling results. This would include guidance on communicating potentially sensitive information.
- Capitalise on the growth in use of patient messaging platforms such as Accurx[47], resulting from the COVID-19 pandemic, to develop formal pathways within General Practices for pro-actively communicating negative results and safety-netting advice:
  - Encourage direct SMS messaging of negative results to patients using pre-built templates which also contain information advising patients to contact their GP again if symptoms persist.
  - For those without a recorded mobile phone number, adopt a receptionist-led system of contacting the patient's landline, sending a letter informing them of their negative result if unable to get through.

Policy

- Instigate policy nationally to support standardisation of practices, such as those suggested above, within primary care.
- Ensure patients are included within the design and evaluation of future services and care models.

Conclusion

Overall, patients presenting in primary care with symptoms of possible CRC find FIT an acceptable test, even when facing challenges to collect their samples. Acceptability was influenced by personal knowledge and previous experience. FIT acceptability was high despite reports of poor experience of care, particularly regarding communication about the test and its results. There is scope for improving the test itself and its communication to enhance patient experience. Future studies should investigate patient acceptability and experience of care among those who choose not to do FIT, across different ethnic groups and different levels of social deprivation.

For peer review only

Figure legend

Figure 1. Themes and subthemes: patient experience and acceptability of using FIT

Acknowledgements

We thank all the patients who agreed to be interviewed. We thank the North East Essex and Suffolk Pathology Services at the East Suffolk and North Essex NHS Foundation Trust, and the Clinical Biochemistry section of Laboratory Medicine at Norfolk and Norwich University Hospitals NHS Foundation Trust. They have added a study recruitment letter, consent form and prepaid envelope to each FIT kit, distributed these to GP practices, and provided data on FIT results. We thank Prof Niek de Wit and Dr Peter Holloway for their support in the earlier stages of the study. We are grateful to Dr Marije van Melle for her work in the early stages of study design. We thank James Brimicombe for data management advice, support and expertise, and Andy Cowan for his help collecting and managing FIT-East study data. We are grateful to the CanTest Patient & Public Involvement (PPI) Panel (special thanks to Margaret Johnson) for reading and commenting on interview transcripts.

Supplemental material

Supplementary File 1. Interview topic schedule
Supplementary File 2. Interviews in context
COREQ checklist

Patient consent for publication

Not required.

Funding statement

This work was supported by the CanTest Collaborative, which is funded by Cancer Research UK grant number C8640/A23385, of which FMW and WH are co-Directors, and NC, MMP and SH are postdoctoral researchers.

This work was co-funded by the National Institute for Health Research (NIHR) Policy Research Programme, conducted through the Policy Research Unit in Cancer Awareness, Screening and Early Diagnosis, PR-PRU-1217-21601. The views expressed in this publication are those of the authors and not necessarily those of the National Health Service, the NIHR or the Department of Health. The funding sources had no role in the study design, data collection, data analysis, data interpretation, writing of the report or in the decision to submit for publication.

Competing interests

None declared.

For peer review only

For peer review only - http://bmjopen.bmj.com/site/about/guidelines.xhtml

Contributors

MMP and FMW planned the original study; WH and FMW secured funding. SH, SA and MMP carried out the interviews. Analysis was planned and carried out by CS, NC, SH, SA, AF, AC and FMW. CS and NC drafted the manuscript with senior input from SA and FMW and revised it after critical reviews from all authors. All authors read and approved the final manuscript.

Data sharing statement

Anonymised data are available upon reasonable request.

Ethics Approval Statement

Ethical approval was granted by the East of England Cambridge Central Research Ethics Committee – reference 19/EE/0036.

For peer review only

REFERENCES

1. Colorectal cancer statistics [Internet]. WCRF International. [cited 2023 Apr 14]. Available from: https://www.wcrf.org/cancer-trends/colorectal-cancer-statistics/

2. 1 Recommendations | Quantitative faecal immunochemical tests to guide referral for colorectal cancer in primary care | Guidance | NICE [Internet]. NICE; [cited 2022 Jan 21]. Available from: https://www.nice.org.uk/guidance/dg30/chapter/1-Recommendations

3. Young GP, Symonds EL, Allison JE, Cole SR, Fraser CG, Halloran SP, et al. Advances in Fecal Occult Blood Tests: The FIT Revolution. Dig Dis Sci. 2015;60(3):609–22.

4. Koo MM, von Wagner C, Abel GA, McPhail S, Hamilton W, Rubin GP, et al. The nature and frequency of abdominal symptoms in cancer patients and their associations with time to help-seeking: evidence from a national audit of cancer diagnosis. J Public Health Oxf Engl. 2018 Sep;40(3):e388–95.

5. Moreno CC, Mittal PK, Sullivan PS, Rutherford R, Staley CA, Cardona K, et al. Colorectal Cancer Initial Diagnosis: Screening Colonoscopy, Diagnostic Colonoscopy, or Emergent Surgery, and Tumor Stage and Size at Initial Presentation. Clin Colorectal Cancer. 2016 Mar;15(1):67–73.

6. Diagnosis | Gastrointestinal tract (lower) cancers - recognition and referral | CKS | NICE [Internet]. [cited 2022 May 19]. Available from: https://cks.nice.org.uk/topics/gastrointestinal-tract-lower-cancers-recognition-referral/diagnosis/

7. Overview | Suspected cancer: recognition and referral | Guidance | NICE [Internet]. NICE; [cited 2022 Jan 21]. Available from: https://www.nice.org.uk/guidance/ng12

8. Hamilton W. The CAPER studies: five case-control studies aimed at identifying and quantifying the risk of cancer in symptomatic primary care patients. Br J Cancer. 2009 Dec 3;101 Suppl 2:S80-86.

9. Bailey SER, Abel GA, Atkins A, Byford R, Davies SJ, Mays J, et al. Diagnostic performance of a faecal immunochemical test for patients with low-risk symptoms of colorectal cancer in primary care: an evaluation in the South West of England. Br J Cancer. 2021 Mar;124(7):1231–6.

10. Pin-Vieito N, Tejido-Sandoval C, Vicente-Bielza N de, Sánchez-Gómez C, Cubiella J. Faecal immunochemical tests safely enhance rational use of resources during the assessment of suspected symptomatic colorectal cancer in primary care: systematic review and meta-analysis. Gut. 2022 May 1;71(5):950–60.

11. The NHS Bowel screening programme | Background information | Bowel screening | CKS | NICE [Internet]. [cited 2022 Feb 1]. Available from: https://cks.nice.org.uk/topics/bowel-screening/background-information/the-nhs-bowel-screening-programme/#bowel-cancer-screening-programme-in-england

12. Joint ACPGBI, BSG and BSGAR considerations for adapting the rapid access colorectoral cancer pathway during COVID-19 pandemic [Internet]. The British Society of

For peer review only

Gastroenterology. 2020 [cited 2022 Jan 24]. Available from: https://www.bsg.org.uk/covid-19-advice/covid-19-advice-for-healthcare-professionals/joint-acgbbi-bsg-and-bsgar-considerations-for-adapting-the-rapid-access-colorectoral-cancer-pathway-during-covid-19-pandemic/

13. Maclean W, Limb C, Mackenzie P, Whyte MB, Benton SC, Rockall T, et al. Adoption of faecal immunochemical testing for 2-week-wait colorectal patients during the COVID-19 pandemic: an observational cohort study reporting a new service at a regional centre. Colorectal Dis. 2021;23(7):1622–9.

14. D'Souza N, Abulafi M. Navigating the storm of COVID-19 for patients with suspected bowel cancer. Br J Surg. 2020 May 13;107(7):e204.

15. Arasaradnam RP, Bhala N, Evans C, Greenaway J, Logan R, Penman I, et al. Faecal immunochemical testing in the COVID-19 era: balancing risk and costs. Lancet Gastroenterol Hepatol. 2020 Aug;5(8):717–9.

16. Faecal Immunochemical Testing (FIT) in patients with signs or symptoms of suspected colorectal cancer (CRC): A joint guideline from the Association of Coloproctology of Great Britain & Ireland (ACPGBI) and the British Society of Gastroenterology (BSG) [Internet]. The British Society of Gastroenterology. [cited 2022 Jun 10]. Available from: https://www.bsg.org.uk/clinical-resource/faecal-immunochemical-testing-fit-in-patients-with-signs-or-symptoms-of-suspected-colorectal-cancer-crc-a-joint-guideline-from-the-acpgbi-and-the-bsg/

17. Monahan KJ, Davies MM, Abulafi M, Banerjea A, Nicholson BD, Arasaradnam R, et al. Faecal immunochemical testing (FIT) in patients with signs or symptoms of suspected colorectal cancer (CRC): a joint guideline from the Association of Coloproctology of Great Britain and Ireland (ACPGBI) and the British Society of Gastroenterology (BSG). Gut [Internet]. 2022 Jul 12 [cited 2022 Aug 8]; Available from: http://gut.bmj.com/content/early/2022/07/25/gutjnl-2022-327985

18. Project information | Quantitative faecal immunochemical tests to guide colorectal cancer pathway referral for people with a change in bowel habit or abdominal pain | Guidance | NICE [Internet]. NICE; [cited 2022 Aug 8]. Available from: https://www.nice.org.uk/guidance/indevelopment/gid-dg10036

19. Sekhon M, Cartwright M, Francis JJ. Acceptability of health care interventions: A theoretical framework and proposed research agenda. Br J Health Psychol. 2018;23(3):519–31.

20. Wagner C von, Verstraete W, Hirst Y, Nicholson BD, Stoffel ST, Laszlo H. Public preferences for using quantitative faecal immunochemical test versus colonoscopy as diagnostic test for colorectal cancer: evidence from an online survey. BJGP Open [Internet]. 2020 Apr 1 [cited 2022 May 27];4(1). Available from: https://bjgpopen.org/content/4/1/bjgpopen20X101007

21. Chambers JA, Callander AS, Grangeret R, O'Carroll RE. Attitudes towards the Faecal Occult Blood Test (FOBT) versus the Faecal Immunochemical Test (FIT) for colorectal cancer screening: perceived ease of completion and disgust. BMC Cancer. 2016 Dec;16(1):1–7.

For peer review only

22. Bowyer HL, Vart G, Kralj-Hans I, Atkin W, Halloran SP, Seaman H, et al. Patient attitudes towards faecal immunochemical testing for haemoglobin as an alternative to colonoscopic surveillance of groups at increased risk of colorectal cancer. J Med Screen. 2013 Sep 1;20(3):149–56.

23. Chin YH, Ng CH, Seah SHY, Jain SR, Law JH, Devi MK, et al. Evolving perspectives on stool testing for colorectal cancer: a qualitative systematic review. Eur J Cancer Prev. 2020 Sep;29(5):416–23.

24. Johanna W, Yvonne W, Anna J, Kaisa F. Two sides of every coin: individuals' experiences of undergoing colorectal cancer screening by faecal immunochemical test and colonoscopy. Eur J Public Health. 2021 Dec 1;31(6):1290–5.

25. Vart G, Banzi R, Minozzi S. Comparing participation rates between immunochemical and guaiac faecal occult blood tests: a systematic review and meta-analysis. Prev Med. 2012 Aug;55(2):87–92.

26. Goodwin B, Viljoen B, Myers L, Ireland M, Dunn J, Chambers S, et al. Identifying modifiable features of home bowel cancer screening kits to facilitate use: consumer perspectives. Public Health Res Pract [Internet]. 2022 [cited 2022 Jun 27]; Available from: https://www.phrp.com.au/?p=41545

27. Delisle TG, D'Souza N, Davies B, Benton S, Chen M, Ward H, et al. Faecal immunochemical test for suspected colorectal cancer symptoms: patient survey of usability and acceptability. BJGP Open [Internet]. 2022 Jan 12 [cited 2022 Feb 8]; Available from: https://bjgpopen.org/content/early/2022/01/10/BJGPO.2021.0102

28. Forster AS, Rubin G, Emery JD, Thompson M, Sutton S, de Wit N, et al. Measuring patient experience of diagnostic care and acceptability of testing. Diagnosis. 2021 Aug 26;8(3):317–21.

29. Gil N, Su H, Kaur K, Barnett M, Murray A, Duffy S, et al. Patient experience and satisfaction with symptomatic Faecal Immunochemical Testing: An explanatory sequential mixed-methods evaluation. Br J Gen Pract [Internet]. 2022 Oct 18 [cited 2022 Oct 19]; Available from: https://bjgp.org/content/early/2022/10/18/BJGP.2022.0241

30. Calanzani N, Pannebakker M, Tagg MJ, Walford H, Holloway P, de Wit N,Hamilton W, Walter FM. Who are the patients being offered the faecal immunochemical test in routine English general practice, and for what symptoms? A prospective descriptive study. BMJ Open 2022. ((in press)).

31. Archer S, Calanzani N, Honey S, Johnson M, Neal R, Scott SE, et al. Impact of the COVID-19 pandemic on cancer assessment in primary care: a qualitative study of GP views. BJGP Open. 2021 Aug;5(4):BJGPO.2021.0056.

32. Tong A, Sainsbury P, Craig J. Consolidated criteria for reporting qualitative research (COREQ): a 32-item checklist for interviews and focus groups. Int J Qual Health Care. 2007 Dec 1;19(6):349–57.

For peer review only - http://bmjopen.bmj.com/site/about/guidelines.xhtml

33. Gale NK, Heath G, Cameron E, Rashid S, Redwood S. Using the framework method for the analysis of qualitative data in multi-disciplinary health research. BMC Med Res Methodol. 2013 Dec;13(1):117.

34. Best Qualitative Data Analysis Software for Researchers | NVivo [Internet]. [cited 2022 May 16]. Available from: https://www.qsrinternational.com/nvivo-qualitative-data-analysis-software/home

35. Scott SE, Walter FM, Webster A, Sutton S, Emery J. The Model of Pathways to Treatment: Conceptualization and integration with existing theory. Br J Health Psychol. 2013;18(1):45–65.

36. Walter F, Webster A, Scott S, Emery J. The Andersen Model of Total Patient Delay: a systematic review of its application in cancer diagnosis. J Health Serv Res Policy. 2012 Apr;17(2):110–8.

37. University of Cambridge | CanTest [Internet]. CanTest |. 2019 [cited 2022 Jun 7]. Available from: https://www.cantest.org/about/university-of-cambridge/

38. Regional ethnic diversity [Internet]. [cited 2022 Jun 23]. Available from: https://www.ethnicity-facts-figures.service.gov.uk/uk-population-by-ethnicity/national-and-regional-populations/regional-ethnic-diversity/latest

39. Vuik FE, Nieuwenburg SA, Bardou M, Lansdorp-Vogelaar I, Dinis-Ribeiro M, Bento MJ, et al. Increasing incidence of colorectal cancer in young adults in Europe over the last 25 years. Gut. 2019 Oct;68(10):1820–6.

40. Araghi M, Soerjomataram I, Bardot A, Ferlay J, Cabasag CJ, Morrison DS, et al. Changes in colorectal cancer incidence in seven high-income countries: a population-based study. Lancet Gastroenterol Hepatol. 2019 Jul 1;4(7):511–8.

41. Loomans-Kropp HA, Umar A. Increasing Incidence of Colorectal Cancer in Young Adults. J Cancer Epidemiol. 2019 Nov 11;2019:9841295.

42. Clarke N, Gallagher P, Kearney PM, McNamara D, Sharp L. Impact of gender on decisions to participate in faecal immunochemical test-based colorectal cancer screening: a qualitative study. Psychooncology. 2016;25(12):1456–62.

43. Litchfield IJ, Bentham LM, Lilford RJ, McManus RJ, Greenfield SM. Patient perspectives on test result communication in primary care: a qualitative study. Br J Gen Pract. 2015 Mar;65(632):e133–40.

44. Sampson R, Cooper J, Barbour R, Polson R, Wilson P. Patients' perspectives on the medical primary–secondary care interface: systematic review and synthesis of qualitative research. BMJ Open. 2015 Oct;5(10):e008708.

45. Banks J, Walter FM, Hall N, Mills K, Hamilton W, Turner KM. Decision making and referral from primary care for possible lung and colorectal cancer: a qualitative study of patients' experiences. Br J Gen Pract. 2014 Dec 1;64(629):e775–82.

For peer review only

46. Skivington K, Matthews L, Simpson SA, Craig P, Baird J, Blazeby JM, et al. A new framework for developing and evaluating complex interventions: update of Medical Research Council guidance. BMJ. 2021 Sep 30;374:n2061.

47. Accurx for Primary Care [Internet]. [cited 2022 Aug 8]. Available from: https://www.accurx.com/primary-care

For peer review only

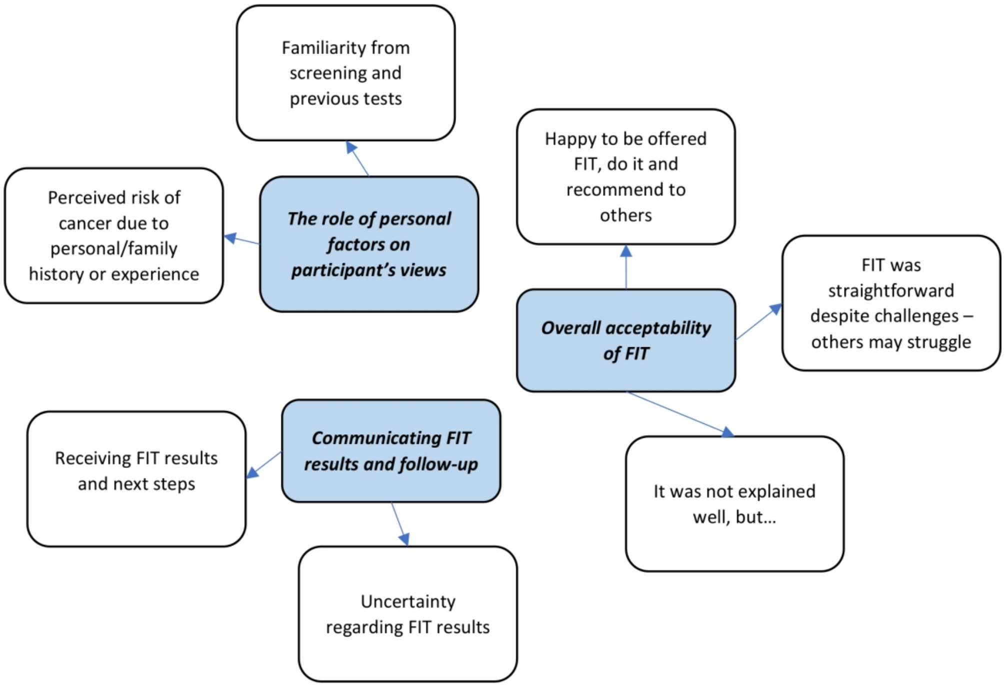

Figure 1. Themes and subthemes: patient experience and acceptability of using FIT

168x114mm (300 x 300 DPI)

Supplementary File 1. Interview topic schedule

Amended COVID items in italics

Background

- Tell me a bit about you:
    - age
    - education
    - employment history
    - daily activities
    - general health

- *What impact has coronavirus had on your everyday life?*

Background to FIT

- What was your overall experience of the problem, getting tested and receiving your test result?

- Tell me more about the symptoms you experienced
    - Abdominal discomfort
    - Change in bowel habit (general, diarrhoea, constipation)
    - Bleeding
    - Indigestion / heartburn
    - Bloating
    - Fatigue
    - Unexplained weight loss
    - Loss of appetite.

- How long had you experienced symptoms?
    - *Note: Try and get dates to be able to work out how this relates to COVID*

- Did you try any self-management?
    - *If yes, do you think coronavirus affected how you self-managed?*

- When did you first seek help for symptoms?
    - *Do you think coronavirus had any impact on when and where you sought help?*
        - *Friends and family*
        - *Other sources of information (e.g. internet)*
        - *Other medical professional (e.g. pharmacist)*
    - *Do you think coronavirus has changed the way you seek help for medical problems?*
        - *Changing thresholds for going to the doctors*
        - *Ongoing fear of going out*
        - *Change in the way that clinical appointments happen*

- Did you take any prescription medication for symptoms?
    - How long taking
    - Any changes to medication and/or dose
    - How medication has helped
    - Any problems?
    - *Do you think coronavirus led to any problems getting medication?*

Experience of GP consultation where the FIT-poo test was first suggested

- How they were feeling before GP appointment (about symptoms, urgency, what did they expect to happen)?

For peer review only

- *Did coronavirus have any impact on how you interacted with your GP?*
    - *Did you have a face to face appointment?*
    - *Were there any remote part to the consultation?*
    - *Was this what you were expecting?*

- *What was it like to going to the GP practice during coronavirus?*
    - *How did this make you feel?*
    - *How do you feel about attending any future appointments with your GP?*

- How did you feel when the GP suggested having a FIT-poo test?
    - How was the FIT-poo test introduced and explained?.
    - Who explained this (GP, nurse, admin?).
    - What did you think about the information you received (format and content).

- How did you feel following the GP consultation but before doing the test (physically and emotionally)?

- How long did you wait between the GP visit and doing the FIT-poo test at home?
    - What factors influenced this (busy/scared/anxious/collecting additional information?).
    - Did you discuss doing the FIT-poo test with anyone (family member, friend, GP, nurse)
    - What was your experience of discussion?
    - Did you look up or request any further information (including internet).
    - Any concerns about taking the test?

- Do you have any ideas about why people might decide against taking the test (why others might be put off)?

- What did you think the doctor was looking for?
    - Did you or they make any links between colorectal cancer and your these symptoms?

Experience of doing the FIT-poo test

- How did you feel about doing the test?
    - Physically
    - Emotionally
    - Did you manage first time you tried? If not, why not?

- How did you feel following the test?
    - Explore for immediately after test
    - Later that day
    - Following day
    - Over following week.
- *Do you think the coronavirus made you more/less concerned about the test?*

Experience of receiving the result of the FIT-poo test

- What was it like waiting for result?
    - *Do you think coronavirus affected this?*
    - For those who have not yet had a result – why do they think this might be?
    - How did you receive the test result (text message, letter, phone call, another consultation)
    - *Do you think coronavirus has had any impact on the timeliness/delivery of results?*

- What was it like being given a POSITIVE / NEGATIVE / EQIVOCAL result?

- Do you know what will happen next?
    - *Are there any concerns about future care because of coronavirus?*

For peer review only

- Do you think your experience has influenced any of your friends or family members about seeking/receiving care now and in the future?

Improvements to the FIT-poo test experience

- What went well before, during and after doing the FIT-poo test?
    - Any suggestions for changes that would improve FIT-poo test
        - Information provided
        - Procedure
        - Staff interpersonal skills
        - Experience of taking the FIT-poo test.

- *What impact, if any, did coronavirus have on your testing experience?*
    - *Has this changed their view of future care and what this might look like post-covid?*

- Would you take test again?

- Would you recommend test to someone else?
    - How would you explain test to someone else?

Ask whether anything else participant would like to mention / discuss

For peer review only

Supplementary File 2. Interviews in context: patients' experiences during the COVID-19 pandemic

Participants described feeling isolated from their support networks due to the limitations on social interactions during the pandemic. This resulted in some feeling unable to have conversations with family and friends regarding their symptoms during the appraisal period.

Many delayed seeking help as a result of what they perceived was happening within General Practice. A range of attitudes were displayed, from believing practices were overrun, to feeling GPs were 'shutdown' or only open for emergencies. For some, this resulted in the decision to seek help from an alternative provider such as 111 or walk-in services. In contrast, for a minority of participants positive media campaigns advising the public that the NHS remained open influenced them to contact their GP.

Changes to remote consulting (primarily in the form of telephone consultations) had both positive and negative impacts. Though many highlighted the convenience of telephone appointments and the speed of talking to their GP, others described the challenges of navigating complicated booking systems, and describing problems over the phone.

For those that visited their GP surgery during the pandemic, personal protective equipment and social distancing measures were generally expected and accepted, despite often feeling time-consuming or frustrating. Many perceived GP practices to be safer and cleaner than other public spaces.

For peer review only

COREQ (COnsolidated criteria for REporting Qualitative research) Checklist

A checklist of items that should be included in reports of qualitative research. You must report the page number in your manuscript where you consider each of the items listed in this checklist. If you have not included this information, either revise your manuscript accordingly before submitting or note N/A.

Topic	Item No.	Guide Questions/Description	Reported on Page No.
**Domain 1: Research team and reflexivity**			
Personal characteristics			
Interviewer/facilitator	1	Which author/s conducted the interview or focus group?	
Credentials	2	What were the researcher's credentials? E.g. PhD, MD	
Occupation	3	What was their occupation at the time of the study?	
Gender	4	Was the researcher male or female?	
Experience and training	5	What experience or training did the researcher have?	
Relationship with participants			
Relationship established	6	Was a relationship established prior to study commencement?	
Participant knowledge of the interviewer	7	What did the participants know about the researcher? e.g. personal goals, reasons for doing the research	
Interviewer characteristics	8	What characteristics were reported about the inter viewer/facilitator? e.g. Bias, assumptions, reasons and interests in the research topic	
**Domain 2: Study design**			
Theoretical framework			
Methodological orientation and Theory	9	What methodological orientation was stated to underpin the study? e.g. grounded theory, discourse analysis, ethnography, phenomenology, content analysis	
Participant selection			
Sampling	10	How were participants selected? e.g. purposive, convenience, consecutive, snowball	
Method of approach	11	How were participants approached? e.g. face-to-face, telephone, mail, email	
Sample size	12	How many participants were in the study?	
Non-participation	13	How many people refused to participate or dropped out? Reasons?	
Setting			
Setting of data collection	14	Where was the data collected? e.g. home, clinic, workplace	
Presence of non-participants	15	Was anyone else present besides the participants and researchers?	
Description of sample	16	What are the important characteristics of the sample? e.g. demographic data, date	
Data collection			
Interview guide	17	Were questions, prompts, guides provided by the authors? Was it pilot tested?	
Repeat interviews	18	Were repeat inter views carried out? If yes, how many?	
Audio/visual recording	19	Did the research use audio or visual recording to collect the data?	
Field notes	20	Were field notes made during and/or after the inter view or focus group?	
Duration	21	What was the duration of the inter views or focus group?	
Data saturation	22	Was data saturation discussed?	
Transcripts returned	23	Were transcripts returned to participants for comment and/or	

For peer review only

Topic	Item No.	Guide Questions/Description	Reported on Page No.
		correction?	
**Domain 3: analysis and findings**			
Data analysis			
Number of data coders	24	How many data coders coded the data?	
Description of the coding tree	25	Did authors provide a description of the coding tree?	
Derivation of themes	26	Were themes identified in advance or derived from the data?	
Software	27	What software, if applicable, was used to manage the data?	
Participant checking	28	Did participants provide feedback on the findings?	
Reporting			
Quotations presented	29	Were participant quotations presented to illustrate the themes/findings? Was each quotation identified? e.g. participant number	
Data and findings consistent	30	Was there consistency between the data presented and the findings?	
Clarity of major themes	31	Were major themes clearly presented in the findings?	
Clarity of minor themes	32	Is there a description of diverse cases or discussion of minor themes?	

Developed from: Tong A, Sainsbury P, Craig J. Consolidated criteria for reporting qualitative research (COREQ): a 32-item checklist for interviews and focus groups. *International Journal for Quality in Health Care*. 2007. Volume 19, Number 6: pp. 349 – 357

Once you have completed this checklist, please save a copy and upload it as part of your submission. DO NOT include this checklist as part of the main manuscript document. It must be uploaded as a separate file.

For peer review only
